# Associations of novel visceral obesity indices (METS-VF and BRI) with dementia: the role of metabolic mediators and genetic susceptibility

**DOI:** 10.3389/fnut.2026.1800772

**Published:** 2026-05-18

**Authors:** Xinrui Xue, Lei Liu, Zhen Tan, Lin Zhou, Shuang Li, Wei Yan, Xinyue Liu, Mao Ye, Xiaoping Li, Hongqiang Ren

**Affiliations:** 1 Department of Cardiology, Suining Central Hospital, Suining, Sichuan, China; 2 Department of Cardiology, Sichuan Provincial People’s Hospital, University of Electronic Science and Technology of China, Chengdu, Sichuan, China

**Keywords:** BRI, dementia, metabolic dysfunction, METS-VF, UK Biobank, visceral obesity

## Abstract

**Background:**

Visceral obesity-related indices may capture metabolically adverse adiposity more effectively than conventional obesity measures, but their comparative associations with dementia risk and the potential roles of metabolic dysfunction and genetic susceptibility remain insufficiently characterized.

**Methods:**

In this prospective cohort study, 327,368 dementia-free participants from the UK Biobank were included. Body roundness index (BRI) and metabolic score for visceral fat (METS-VF) were first validated against imaging-derived visceral fat measures and then examined for their associations with incident all-cause dementia, Alzheimer’s disease (AD), and vascular dementia using Cox proportional hazards models and restricted cubic spline analyses. Additional analyses assessed subgroup effects, sensitivity, multicollinearity, incremental explanatory value beyond BMI and waist circumference (WC), metabolomic mediation, genetic susceptibility, and longitudinal changes in anthropometric and metabolic markers before dementia diagnosis.

**Results:**

During follow-up, 8,768 participants developed dementia. METS-VF and BRI showed the strongest correlations with imaging-derived visceral adiposity among the tested obesity indices. Higher METS-VF levels were associated with increased risks of all-cause dementia, vascular dementia, and AD, with significant nonlinear threshold effects. BRI was also positively associated with all three dementia outcomes, although its association with AD was attenuated after full adjustment but remained statistically significant. The association between METS-VF and AD was particularly evident among individuals with low to intermediate genetic risk. Exploratory mediation analyses suggested that metabolic dysfunction may partly explain the observed associations between visceral obesity indices and dementia-free survival time. Among participants who subsequently developed dementia, WC and fasting blood glucose increased before diagnosis, whereas BMI remained relatively stable.

**Conclusion:**

Visceral obesity, particularly as captured by METS-VF, was independently associated with increased dementia risk. These findings support moving beyond BMI-centric obesity definitions and highlight visceral adiposity-related metabolic dysfunction as a potential focus for dementia risk stratification and prevention research.

## 1 Introduction

The global prevalence of dementia is increasing rapidly due to population aging, with projections indicating an increase from 57.4 million in 2019 to 152.8 million by 2050 (1). Dementia imposes significant health and economic burdens worldwide (2). Despite these challenges, effective treatments for dementia remain limited. Therefore, identifying modifiable risk factors for dementia is critical for the development of preventive and therapeutic strategies. While some studies have suggested that obesity could be a modifiable risk factor for dementia (3, 4), others have highlighted a phenomenon known as the “obesity paradox,” where obese individuals seem to have a lower risk of dementia (5–7). This paradox may largely reflect reverse causation. In the prodromal phase of neurodegenerative diseases, pathological changes in brain regions regulating appetite and metabolism may lead to progressive weight loss years before clinical diagnosis (8, 9). Consequently, individuals with normal or low body weight at the time of assessment may already be experiencing disease-related metabolic decline, which can create the misleading impression that obesity is protective. Recent work has highlighted the importance of considering the temporal relationship between adiposity measurement and dementia onset when interpreting these associations (10).

Most previous studies have defined obesity using the Body Mass Index (BMI), which does not distinguish between visceral and subcutaneous fat (11), and its utility in evaluating body adipose tissue distribution is limited (12). This limitation makes BMI less effective for assessing obesity-related health risks, including those associated with dementia.

Visceral adipose tissue (VAT) has been more strongly linked to metabolic risk factors such as fasting glucose, triglycerides (TG), high-density lipoprotein cholesterol (HDL-C), and metabolic syndrome (13), and a potential link between VAT and dementia has been proposed (14). However, despite growing evidence, the relationship between visceral fat and dementia remains incompletely characterized.

Current methods for measuring VAT, such as abdominal computed tomography (CT) and magnetic resonance imaging (MRI), are expensive and not suitable for large-scale population studies (15). To address this, several novel indices have been developed to estimate visceral obesity, including BRI (16), METS-VF (17), visceral adiposity index (VAI) (18), and lipid accumulation product (LAP) (19). Notably, a meta-analysis involving more than five million participants showed that central obesity was associated with an increased risk of cognitive impairment and dementia (7). Nonetheless, two important gaps remain. First, most large-scale studies have relied on simple anthropometric markers such as WC or waist-to-hip ratio, whereas composite indices such as METS-VF and BRI may better capture visceral adiposity together with its metabolic consequences. Second, few studies have validated these indices against imaging-derived VAT, directly compared them with conventional obesity measures, or simultaneously examined their associations with incident dementia, metabolic profiles, and genetic susceptibility within a single cohort.

In addition, recent evidence regarding visceral adiposity-related indices and cognition has been inconsistent. For example, higher VAI was associated with a lower risk of cognitive impairment in a recent CHARLS study, a finding that may reflect reverse causation (20). In contrast, higher METS-VF and BRI have been linked to poorer cognitive outcomes, and LAP has shown sex-specific prospective associations (20–22). However, these studies were mostly cross-sectional, focused on cognitive performance rather than incident dementia, and generally lacked imaging validation and mechanistic investigation. Therefore, the long-term associations between visceral obesity indices and incident dementia, as well as the underlying metabolic and genetic mechanisms, remain insufficiently characterized.

This study aimed to investigate the associations between novel visceral obesity indices and incident dementia in the UK Biobank and to explore potential metabolic and genetic modifiers of these associations.

## 2 Materials and methods

### 2.1 Study design and participants

The UK Biobank is a large-scale prospective cohort study that enrolled approximately 500,000 participants aged 37–73 years from 22 assessment centers across the United Kingdom. Baseline data were collected between 2006 and 2010, with three subsequent follow-up assessments: the first from 2012 to 2013, the second from 2014 onward, and the third beginning in 2019. These assessments included touchscreen questionnaires, verbal interviews, physical measurements, biological sampling, and linkage to electronic health records (23). In the present study, baseline demographic characteristics, anthropometric measures, biochemical indicators, and lifestyle-related variables were extracted from the initial assessment visit conducted between 2006 and 2010. Repeated measurements of visceral obesity indices used in the trajectory analyses, including the second measurements of BRI and METS-VF, were obtained from the first repeat assessment visit conducted between 2012 and 2013. Imaging-derived visceral fat data, including MRI-measured visceral fat volume and DXA-measured visceral fat mass, were extracted from the imaging assessment visits conducted from 2014 onward. The UK Biobank was approved by the North West Multi-Centre Research Ethics Committee, and this study was conducted under the UK Biobank Application ID: 106027.

Of the initial 502,128 participants, 327,368 were included in the present analysis after excluding those who (1) had dementia at baseline; (2) had incomplete data required to calculate BRI, LAP, METS-VF, and VAI; or (3) were younger than 50 years.

Participants missing MRI data used to assess visceral fat volume or missing DXA imaging data used to evaluate visceral fat mass were excluded from the corresponding analyses, resulting in subsamples of 34,831 and 25,109 participants, respectively. These subsamples were used to assess the validity of visceral obesity indices against MRI- and DXA-measured visceral fat mass and volume.

**FIGURE 1:**
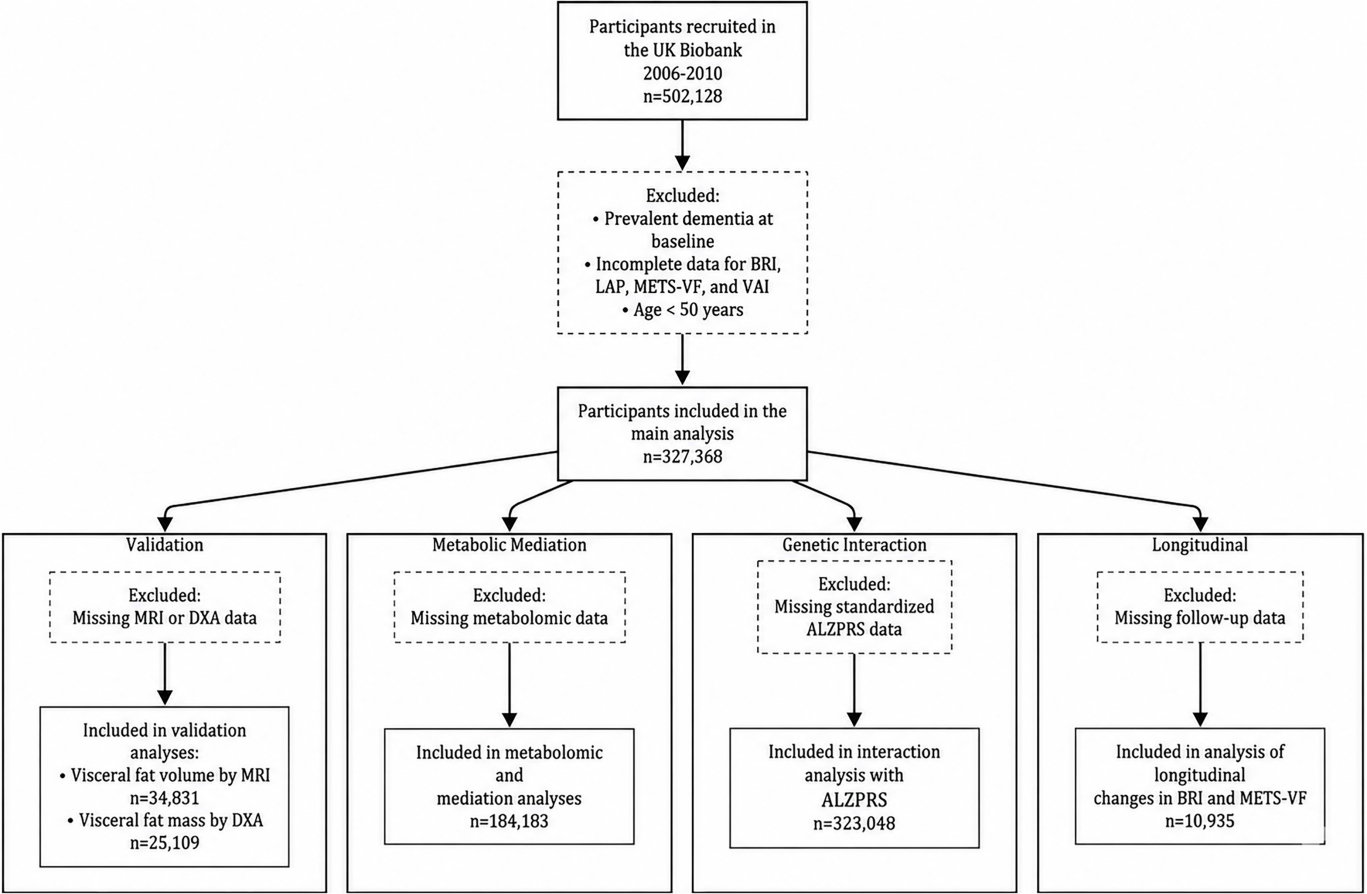
Flowchart of participant selection in the UK Biobank. Flow diagram illustrating participant selection for the present study. Of the 502,128 UK Biobank participants, those with prevalent dementia at baseline, missing data for visceral obesity indices, or age < 50 years were excluded. Subsamples with available MRI, DXA, PRS, and metabolomic data were identified for specific analyses.

Participants missing standardized AD polygenic risk score (ALZPRS) data or metabolomic data were excluded from specific analyses, yielding 323,048 participants for PRS interaction analyses and 184,183 for metabolomic and mediation analyses. A flowchart outlining participant selection is shown in Figure 1.

### 2.2 Visceral obesity indices

In this study, we analyzed four visceral obesity indices: METS-VF, BRI, LAP, and VAI. These indices rely on anthropometric measurements such as waist circumference (WC) and height. Levels of TG, fasting blood glucose (FBG), and HDL-C were used in their calculations. BMI was calculated as weight (kg) divided by height squared (m^2^).

METS-VF was calculated using a previously established formula (17), which requires two intermediate variables: waist-to-height ratio (WHTR) and Metabolic Score for Insulin Resistance (METS-IR). The specific calculation steps were:


$$W\,H\,T\,R=\frac{W\,C}{H\,e\,i\,g\,h\,t}$$

$$M\,E\,T\,S-I\,R=\frac{l\,n\,\left(2\times F\,B\,G+T\,G\right)\times B\,M\,I}{l\,n\,\left(H\,D\,L\right)}$$

$$METS-VF=4.466+0.01\times {\left(\text{ln}\left(METS-IR\right)\right)}^{3}+3.329\times$$

$${\left(l\,n\,\left(W\,H\,T\,R\right)\right)}^{3}+0.319\times g\,e\,n\,d\,e\,r+0.594\times l\,n\,\left(a\,g\,e\right)$$

The formula for BRI was obtained from previous literature (24), with calculations as follows:


$$B\,R\,I=364.2-365.5\times \sqrt{1-{\left(\frac{W\,C\,\left(c\,m\right)/2\,\pi }{0.5\times h\,e\,i\,g\,h\,t\,\left(c\,m\right)}\right)}^{2}}$$

LAP was calculated with sex-specific formulas (25), given its known sex-related differences in clinical interpretation:


F⁢e⁢m⁢a⁢l⁢e⁢L⁢A⁢P=[W⁢C⁢(c⁢m)-58]×T⁢G⁢(m⁢m⁢o⁢l/L)

M⁢a⁢l⁢e⁢L⁢A⁢P=[W⁢C⁢(c⁢m)-65]×T⁢G⁢(m⁢m⁢o⁢l/L)

VAI also adopts sex-specific coefficients to account for sex differences in adipose distribution (18), with the formula:


$$F\,e\,m\,a\,l\,e\,V\,A\,I=\left(\frac{W\,C\,\left(c\,m\right)}{36.58+1.89\times B\,M\,I\,\left(k\,g/{\text{m}}^{2}\right)}\right)$$

$$\times \left(\frac{T\,G\,\left(m\,m\,o\,l/L\right)}{0.81}\right)\times \left(\frac{1.52}{H\,D\,L\,\left(m\,m\,o\,l/L\right)}\right)$$

$$M\,a\,l\,e\,V\,A\,I=\left(\frac{W\,C\,\left(c\,m\right)}{39.68+1.88\times B\,M\,I\,\left(k\,g/{\text{m}}^{2}\right)}\right)$$

$$\times \left(\frac{T\,G\,\left(m\,m\,o\,l/L\right)}{1.03}\right)\times \left(\frac{1.31}{H\,D\,L\,\left(m\,m\,o\,l/L\right)}\right)$$

### 2.3 Definitions of outcomes

The outcomes of this study included all-cause dementia and its two primary subtypes: AD and vascular dementia. Participants with other dementia subtypes were not analyzed separately because of the heterogeneity of these disease entities and the relatively small sample sizes for each subtype, which were insufficient for independent analysis. Dementia outcomes were defined using the International Classification of Diseases, 10th Revision (ICD-10) codes as follows: all-cause dementia (F00, F01, F02, F03, F05.1, G30, G31.1, G31.8), AD (F00, G30), and vascular dementia (F01). Follow-up duration was defined as the time from baseline to the first recorded dementia diagnosis, loss to follow-up (i.e., censoring), or the last available follow-up date, whichever occurred first.

### 2.4 Calculation of polygenic risk score

In the UK Biobank, polygenic risk score (PRS) were calculated as the weighted sum of the effect sizes of individual genetic variants multiplied by their respective allele dosages, using a Bayesian approach applied to meta-analyzed summary statistics from genome-wide association study (GWAS) data (26, 27). Because standardized PRS were available only for specific diseases in the UK Biobank, our analysis was restricted to the AD PRS. In this study, AD PRS were categorized into three groups: low (first quintile), intermediate (second to fourth quintiles), and high (fifth quintile). This categorization emphasizes the contrast between the lowest- and highest-risk groups while maintaining sufficient sample size for the intermediate-risk group.

### 2.5 Metabolomic biomarkers

High-throughput nuclear magnetic resonance (NMR) spectroscopy-based metabolomic profiling was used to quantify 249 metabolic biomarkers (168 directly measured biomarkers and 81 ratios) from ethylenediaminetetraacetic acid (EDTA) plasma samples of approximately 280,000 UK Biobank participants. Detailed descriptions of the NMR metabolomics protocol have been reported previously (28). All metabolite concentrations were transformed using the natural logarithm [ln(X + 1)] and then standardized to z-scores to facilitate comparability across metabolites.

### 2.6 Ascertainment of covariates

The covariates incorporated in this study encompassed six major domains: (1) Sociodemographic characteristics: including age, sex, ethnicity, educational attainment, and the Townsend Deprivation Index (TDI); (2) Physical measurements: including WC, height, systolic blood pressure (SBP), diastolic blood pressure (DBP), and pulse rate; (3) Lifestyle behaviors: including smoking status, alcohol consumption, dietary pattern, physical activity level, and sleep duration; (4) Clinical diagnoses: covering hypertension and diabetes; (5) Biochemical parameters: encompassing FBG, TG, HDL-C, low-density lipoprotein cholesterol (LDL-C), glycated hemoglobin (HbA1c), uric acid (UA), blood urea nitrogen (BUN), and estimated glomerular filtration rate (eGFR); and (6) Medication use: including insulin, aspirin, antihypertensive agents, and lipid-lowering medications. TDI (29), an area-based socioeconomic measure derived from participants’ residential postal codes, was also included to adjust for regional socioeconomic confounding. Educational attainment was stratified into two categories based on whether participants had obtained a college or university degree; smoking and alcohol consumption status were self-reported by participants, with each categorized into three groups: never, previous, and current. Dietary patterns were classified as “healthy” or “unhealthy” based on the consumption of fruits, vegetables, fish, processed meats, and unprocessed red meats. A detailed overview can be found in Supplementary Table S1. Physical activity level was quantified by weekly minutes of moderate-to-vigorous physical activity, and the level was categorized into low, moderate, and high (30). Hypertension was defined by either hospital diagnosis, use of antihypertensive medications, and/or blood pressure ≥ 140/90 mmHg (31). Diabetes was defined as self-reported physician-diagnosed diabetes, current use of hypoglycemic medications, FBG ≥ 7 mmol/L, and/or HbA1c ≥ 6.5% at baseline (32). Biochemical indicators (TG, HDL-C, LDL-C, HbA1c, UA, BUN) were measured using standardized blood testing protocols to ensure data consistency and reliability. The eGFR is calculated by the Chronic Kidney Disease Epidemiology Collaboration (CKD-EPI) equations (33).

### 2.7 Statistical analysis

#### 2.7.1 Description of the basic characteristics

All statistical analyses were conducted using R software (version 4.3.0; Institute of Statistics and Mathematics, University of Vienna, Austria). The normality of continuous variables was assessed using the Anderson-Darling test. Continuous variables were summarized as mean ± standard deviation (SD) for normally distributed data. For non-normally distributed variables, data were presented as median (interquartile range, IQR) [M (P25, P75)]. Differences across groups were assessed using one-way analysis of variance (ANOVA) for normally distributed data and the Kruskal–Wallis test for comparisons among three or more groups. Categorical variables were reported as frequencies (percentages), and Pearson’s Chi-square tests were conducted to compare proportions across groups (Fisher’s exact test was used if expected cell frequencies were < 5). Missing covariate values were handled using multiple imputation.

#### 2.7.2 Association analysis

Linear regression analyses were used to examine the relationships between the visceral obesity indices (METS-VF, BRI, LAP, VAI) and both visceral fat volume (measured by MRI) and fat mass (measured by DXA). To avoid overestimating model fit due to a large number of variables, we reported adjusted R-squared (adjusted R^2^) values to evaluate the goodness-of-fit. Model parameters were assessed for predictive precision using 95% confidence intervals (95% CI).

Receiver operating characteristic (ROC) curves were constructed to evaluate the predictive performance of six obesity indices (METS-VF, BRI, LAP, VAI, BMI, and WC) for 10-year incident all-cause dementia, AD, and vascular dementia. The time-dependent area under the curve (TD-AUC) was calculated to compare the predictive performance of these indices. Through these analyses, we identified METS-VF and BRI as the two indices with the highest predictive efficacy.

Cumulative incidence curves for all-cause dementia, AD, and vascular dementia were estimated using the Kaplan-Meier method. Log-rank tests were used to compare differences across quartile groups of METS-VF and BRI.

To examine the associations of METS-VF and BRI with dementia outcomes, we constructed three Cox proportional hazards regression models. The proportional hazards assumption was assessed using Schoenfeld residuals, and no significant violations were detected. Hazard ratios (HRs) and 95% CI were obtained from the following models: Model 1, Unadjusted; Model 2, Adjusted for age, sex, ethnicity, TDI, educational level, smoking status, alcohol consumption; Model 3, Further adjusted for dietary pattern, physical activity level, sleep duration, UA, eGFR, insulin use, aspirin use, antihypertensive drug use, and cholesterol-lowering medications. To avoid potential multicollinearity, METS-VF and BRI were entered separately in Model 3. Multicollinearity among variables in Model 3 was assessed using the generalized variance inflation factor (GVIF), with GVIF < 2 indicating no evidence of multicollinearity.

Potential nonlinear associations between METS-VF/BRI and dementia outcomes were examined using restricted cubic spline (RCS) regression models with 3 knots placed at the 10th, 50th, and 90th percentiles of the METS-VF/BRI distributions. The spline models were adjusted for the same covariates included in Model 3. To further characterize potential threshold effects, candidate inflection points were identified from the fitted RCS curves using the RCSSCI package. After a candidate threshold was identified, two-piecewise Cox proportional hazards regression models were fitted on either side of the threshold, with adjustment for all covariates included in Model 3. Likelihood ratio tests were used to compare the fit of the linear model with that of the threshold model; a two-piecewise model with P < 0.05 was considered to indicate evidence of a significant threshold effect.

#### 2.7.3 Polygenic risk score

To evaluate the joint effects of PRS and METS-VF/BRI on dementia risk, stratified analyses were performed based on genetic risk categories (low, intermediate, and high). Within each genetic risk category, participants were further grouped into four quartiles (Q1, Q2, Q3, Q4) based on the levels of METS-VF and BRI, using the 25th, 50th, and 75th percentiles as cutoffs.

The associations between METS-VF/BRI and dementia outcomes within each genetic risk stratum were analyzed using multivariate Cox proportional hazards regression Model 3.

#### 2.7.4 Interaction analysis

Interaction analyses were conducted to assess the joint effects of METS-VF/BRI and PRS, with both multiplicative and additive interactions evaluated. For multiplicative interactions, the product term of METS-VF/BRI and PRS was incorporated into Model 3, and the hazard ratio (HR) with its 95% CI for the product term was used to determine statistical significance. For additive interaction, three indices were calculated, including the relative excess risk due to interaction (RERI), attributable proportion (AP), and synergy index (SI). A synergistic additive interaction was indicated by RERI > 0, AP > 0, and SI > 1, whereas antagonistic effects were suggested by RERI < 0, AP < 0, and SI < 1. These indices, together with their 95% CI, were computed using the delta method.

#### 2.7.5 Mediation analysis

Mediation analyses were conducted to evaluate whether the metabolic risk score (MRS) mediates the associations of visceral adiposity indices (METS-VF and BRI) with time free of all-cause dementia, AD, and vascular dementia. Before modeling, metabolite concentrations with extreme values (below the 1st or above the 99th percentile) were handled using a capping (winsorization) approach. All 249 metabolites were then transformed using a natural logarithm [ln(x + 1)] and standardized to z-scores.

To construct the MRS, all 249 processed plasma metabolites were entered into least absolute shrinkage and selection operator Cox regression (LASSO-Cox) models with 10-fold cross-validation, using dementia incidence as the survival outcome. The optimal subset of metabolites was selected using the lambda.1se criterion, corresponding to the most parsimonious model within one standard error of the minimum cross-validated error. The MRS was then calculated as a weighted sum of the selected metabolites, with the corresponding LASSO-Cox regression coefficients used as weights.

The mediation analysis was performed in three steps. First, linear regression models were used to examine the associations of the exposure variables (METS-VF and BRI) with the mediator (MRS). Second, parametric survival regression models were fitted to simultaneously assess the associations of the exposure variables (METS-VF and BRI) and the mediator (MRS) with dementia-free survival time. Third, mediation analyses were implemented using the R package mediation with 1,000 simulations to estimate the direct effect, indirect effect, total effect, and the proportion mediated by MRS in the associations of METS-VF and BRI with dementia-free survival time.

#### 2.7.6 Subgroup and sensitivity analyses

Subgroup and sensitivity analyses were performed to assess the robustness of the main findings and to examine potential effect modification. Subgroup analyses were conducted according to age, sex, ethnicity, educational level, smoking status, alcohol consumption, dietary pattern, physical activity level, and sleep duration. HRs and 95% CI were estimated for each 1-standard deviation increase in METS-VF and BRI, and interaction tests were performed to evaluate potential effect modification across subgroups.

Several sensitivity analyses were also conducted. First, to account for the potential competing effect of death, competing risk models were fitted with all-cause mortality treated as a competing event. Second, to mitigate potential reverse causation, participants who were diagnosed with incident dementia within the first 5 years of follow-up were excluded, and the analyses were repeated using the fully adjusted Cox proportional hazards model (Model 3). Third, because BMI may be susceptible to reverse causation due to preclinical disease-related weight loss, participants with underweight at baseline (BMI < 18.5 kg/m^2^) were excluded, and the analyses were repeated using Model 3. Fourth, in the trajectory analyses of METS-VF and BRI, to further reduce potential prodromal reverse causation, participants who developed incident dementia within 5 years after the second follow-up were excluded, and the trajectory analyses were repeated.

## 3 Results

### 3.1 Participant characteristics

Among the 327,368 eligible participants, 8,768 (2.7%) developed dementia during a median follow-up of 15.2 years. Baseline characteristics differed significantly between participants who developed dementia and those who did not (Table 1). Individuals with dementia were older, more likely to be male, had lower height, higher SBP but lower DBP, and were less likely to have attained a higher education level. They were also more likely to be smokers, non-drinkers, and to report a relatively healthy dietary pattern.

**TABLE 1:** Characteristics of study participants according to incident dementia status.

Characteristics	Overall (*n* = 327,368)	Non-dementia (*n* = 318,600)	Incident dementia (n = 8,768)	*P*-value
Age, y, mean (SD)	60.09 ± 5.45	59.96 ± 5.43	64.52 ± 4.11	< 0.001
Sex, n (%)				
Male	151,353 (46.23)	146,772 (46.07)	4,581 (52.25)	< 0.001
Female	176,015 (53.77)	171,828 (53.93)	4,187 (47.75)	
Ethnicity, n (%)				
White	312,924 (95.59)	304,522 (95.58)	8,402 (95.83)	
Other	14,444 (4.41)	14,078 (4.42)	366 (4.17)	0.283
Waist, cm, mean (SD)	91.03 ± 13.37	90.98 ± 13.35	92.92 ± 13.73	< 0.001
Height, cm, mean (SD)	168.07 ± 9.23	168.09 ± 9.23	167.25 ± 9.20	< 0.001
SBP, mmHg, mean (SD)	140.48 ± 18.64	140.38 ± 18.61	144.20 ± 19.23	< 0.001
DBP, mmHg, mean (SD)	82.62 ± 10.06	82.64 ± 10.05	81.83 ± 10.26	< 0.001
Pulse, bpm, median (IQR)	68.5 (61.5–76)	68.5 (61.5–76)	69 (61.5–77)	< 0.001
Education, n (%)				
College degree or above	98,221 (30.19)	96,421 (30.46)	1,800 (20.63)	
Other	227,089 (69.81)	220,165 (69.54)	6,924 (79.37)	< 0.001
Smoking status, n (%)				
Never	171,583 (52.69)	167,536 (52.85)	4,047 (46.64)	< 0.001
Former	123,067 (37.79)	119,326 (37.64)	3,741 (43.11)	
Current	31,007 (9.52)	30,118 (9.50)	889 (10.25)	
Alcohol status, n (%)				
Never	14,116 (4.32)	13,526 (4.25)	590 (6.76)	< 0.001
Former	12,113 (3.71)	11,553 (3.63)	560 (6.41)	
Current	300,417 (91.97)	292,837 (92.11)	7,580 (86.83)	
Diet, n (%)				
Unhealthy	186,671 (57.19)	181,973 (57.28)	4,698 (53.86)	< 0.001
Healthy	139,758 (42.81)	135,734 (42.72)	4,024 (46.14)	
Physical activity, n (%)				
Low	38,799 (12.56)	37,689 (12.52)	1,110 (13.96)	< 0.001
Moderate	67,781 (21.94)	66,361 (22.05)	1,420 (17.86)	
High	202,349 (65.50)	196,930 (65.43)	5,419 (68.17)	
Sleep duration, n (%)				
< 7 h	80,929 (24.91)	78,690 (24.88)	2,239 (25.92)	<0.001
7–8 h	217,381 (66.90)	212,013 (67.03)	5,368 (62.14)	
>8 h	26,636 (8.20)	25,604 (8.09)	1,032 (11.95)	
Diabetes, n (%)				
NO	300,294 (91.73)	292,959 (91.95)	7,335 (83.66)	< 0.001
YES	27,074 (8.27)	25,641 (8.05)	1,433 (16.34)	
Hypertension, n (%)				
NO	129,209 (39.47)	126,901 (39.83)	2,308 (26.32)	< 0.001
YES	198,159 (60.53)	191,699 (60.17)	6,460 (73.68)	
Cholesterol-lowering medication, n (%)				
NO	256,934 (78.56)	251,373 (78.97)	5,561 (63.53)	< 0.001
YES	70,123 (21.44)	66,931 (21.03)	3,192 (36.47)	
Blood pressure medication, n (%)				
NO	244,208 (74.67)	238,904 (75.06)	5,304 (60.60)	< 0.001
YES	82,849 (25.33)	79,400 (24.94)	3,449 (39.40)	
Insulin, n (%)				
NO	323,181 (98.81)	314,752 (98.88)	8,429 (96.30)	< 0.001
YES	3,876 (1.19)	3,552 (1.12)	324 (3.70)	
Aspirin, n (%)				
NO	272,830 (83.42)	266,537 (83.74)	6,293 (71.90)	< 0.001
YES	54,233 (16.58)	51,773 (16.26)	2,460 (28.10)	
TDI	−2.28 (−3.71 to 0.25)	−2.29 (−3.71 to 0.23)	−1.96 (−3.55 to 0.96)	< 0.001
Obesity indices				
METS-VF, mean (SD)	6.73 ± 0.61	6.73 ± 0.61	6.85 ± 0.60	< 0.001
BRI, median (IQR)	4.07 (3.18–5.12)	4.06 (3.16–5.11)	4.35 (3.41–5.48)	< 0.001
VAI, median (IQR)	1.72 (1.09–2.75)	1.72 (1.09–2.75)	1.78 (1.12–2.85)	< 0.001
LAP, median (IQR)	44.36 (25.23–74.46)	44.3 (25.17–74.36)	46.62 (27.28–78.45)	< 0.001
BMI, kg/m^2^, median (IQR)	26.90 (24.33–30.02)	26.89 (24.33–30.02)	27.15 (24.46–30.27)	< 0.001
Biochemical indicators, median (IQR)				
FBG, mmol/L	4.97 (4.639–5.365)	4.968 (4.638–5.36)	5.063 (4.691–5.557)	< 0.001
TG, mmol/L	1.53 (1.09–2.19)	1.53 (1.09–2.19)	1.56 (1.10–2.19)	0.415
HDL-C, mmol/L	1.41 (1.18–1.69)	1.41 (1.18–1.69)	1.37 (1.13–1.66)	< 0.001
LDL-C, mmol/L	3.57 (2.97–4.17)	3.57 (2.98–4.18)	3.38 (2.73–4.08)	< 0.001
HbA1c, %	5.42 (5.20–5.66)	5.42 (5.20–5.65)	5.51 (5.27–5.80)	< 0.001
UA, μmol/L	307.20 (255.70–363.90)	307.00 (255.60–363.80)	312.25 (259.50–367.60)	< 0.001
BUN, μmol/L	5.41 (4.64–6.29)	5.41 (4.64–6.28)	5.55 (4.71–6.54)	< 0.001
eGFR, mL/min/1.73 m^2^	87.76 (78.59–96.29)	87.89 (78.76–96.39)	83.08 (73.10–92.06)	< 0.001
MRI-measured VAT volume, cm^3^	3.68 (2.26–5.51)	3.68 (2.26–5.51)	3.61 (2.42–5.46)	0.948
DXA-measured VAT mass, g	1075.00 (544.00–1796.00)	1076.00 (544.00–1797.75)	1016.00 (575.00–1654.00)	0.654

Baseline demographic characteristics, anthropometric measures, biochemical indicators, lifestyle-related variables, and baseline visceral obesity indices in Table 1 were extracted from the initial assessment visit (2006–2010). Imaging-derived visceral adiposity measures, including MRI-measured visceral adipose tissue volume and DXA-measured visceral adipose tissue mass, were obtained from the imaging assessment visits conducted from 2014 onward. SBP, systolic blood pressure; DBP, diastolic blood pressure; TDI, Townsend Deprivation Index; METS-VF, Metabolic Score for Visceral Fat; BRI, Body Roundness Index; LAP, Lipid Accumulation Product; VAI, Visceral Adiposity Index; BMI, Body Mass Index; FBG, Fasting Blood Glucose; TG, Triglycerides; HDL-C, High-Density Lipoprotein Cholesterol; LDL-C, Low-Density Lipoprotein Cholesterol; HbA1c, Glycated Hemoglobin; UA, Uric Acid; BUN, Blood Urea Nitrogen; eGFR, Estimated Glomerular Filtration Rate.

Clinically, participants with dementia exhibited a higher prevalence of hypertension and diabetes and were more frequently treated with antihypertensive, lipid-lowering medications, aspirin, and insulin. They also had higher levels of pulse rate, FBG, TG, HbA1c, UA, and BUN, along with lower LDL-C and HDL-C levels. Importantly, all visceral obesity indices were elevated in the dementia group, indicating a higher prevalence of visceral adiposity.

### 3.2 Validation and predictive performance of visceral obesity indices

**FIGURE 2:**
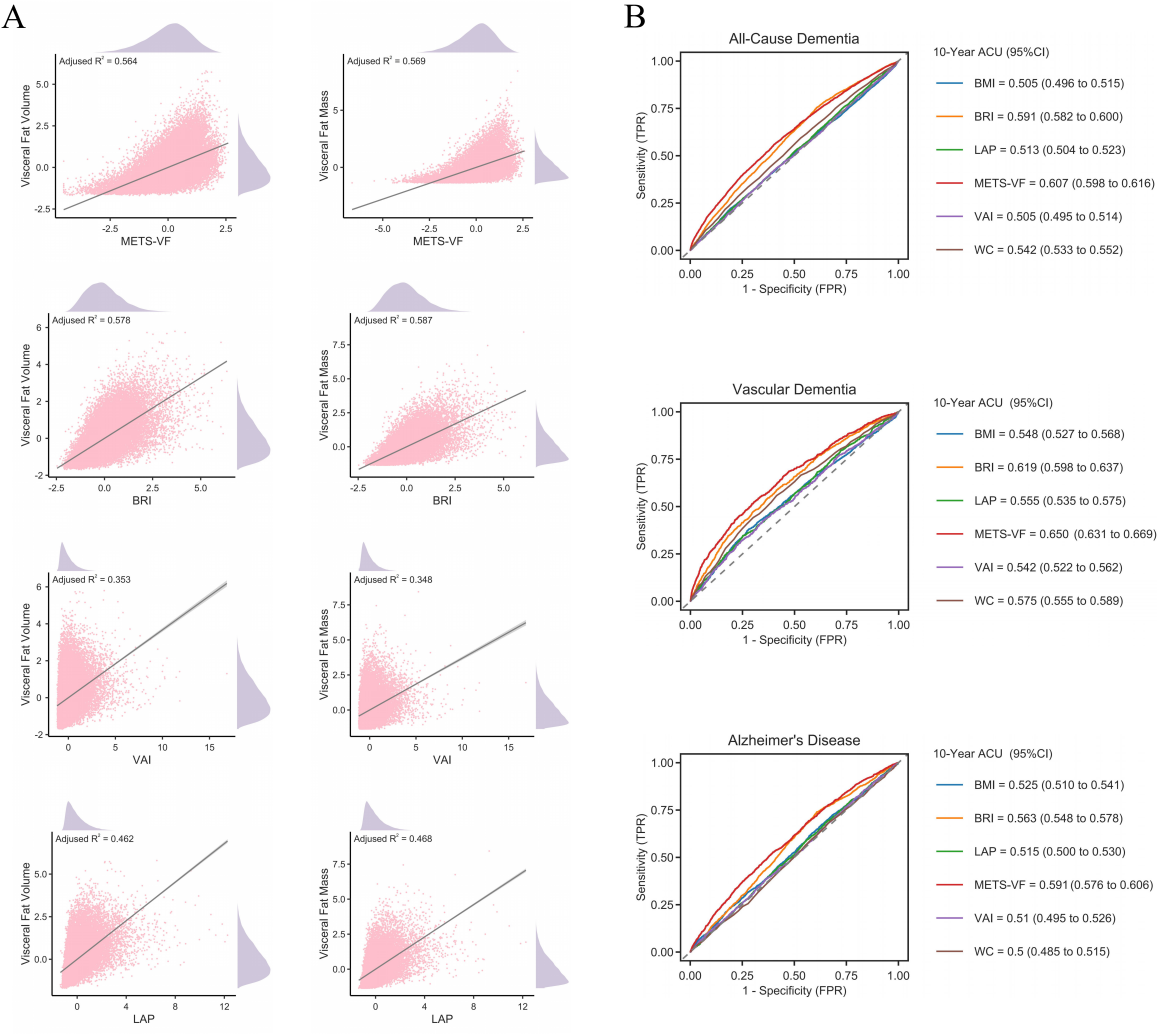
Validation and predictive performance of visceral obesity indices. **(A)** Associations of METS-VF, BRI, VAI, and LAP with MRI-measured visceral fat volume and DXA-measured visceral fat mass. Adjusted R^2^ values are shown in each subpanel. **(B)** Ten-year time-dependent receiver operating characteristic curves for all-cause dementia, vascular dementia, and Alzheimer’s disease. The corresponding area under the curve values for BMI, BRI, LAP, METS-VF, VAI, and WC are shown in each subpanel.

The associations between visceral obesity indices and imaging-derived visceral adiposity, as well as their predictive performance for dementia outcomes, are shown in Figure 2. In the MRI subset (*n* = 34,831) and DXA subset (*n* = 25,109), METS-VF and BRI exhibited the strongest correlations with MRI-measured visceral fat volume and DXA-measured visceral fat mass. For visceral fat volume, BRI demonstrated the highest explanatory power (adjusted R-squared = 0.578), followed closely by METS-VF (adjusted R-squared = 0.564). A similar pattern was observed for visceral fat mass, where BRI again showed the strongest association (adjusted R-squared = 0.587), with METS-VF performing comparably well (adjusted R-squared = 0.569).

ROC analyses showed that METS-VF and BRI performed better than traditional obesity indices, including BMI, LAP, VAI, and WC, in predicting incident dementia. METS-VF yielded the highest 10-year TD-AUC values for all outcomes, with 0.607 (95% CI, 0.598–0.616) for all-cause dementia, 0.650 (95% CI, 0.631–0.669) for vascular dementia, and 0.591 (95% CI, 0.576–0.606) for AD. BRI also demonstrated moderate discriminative ability, with TD-AUC values of 0.591 (95% CI, 0.582–0.600) for all-cause dementia, 0.619 (95% CI, 0.598–0.637) for vascular dementia, and 0.563 (95% CI, 0.548–0.578) for AD. Based on these validation and discrimination results, subsequent association and mechanistic analyses focused on METS-VF and BRI.

### 3.3 Associations of METS-VF and BRI with dementia risk

**FIGURE 3:**
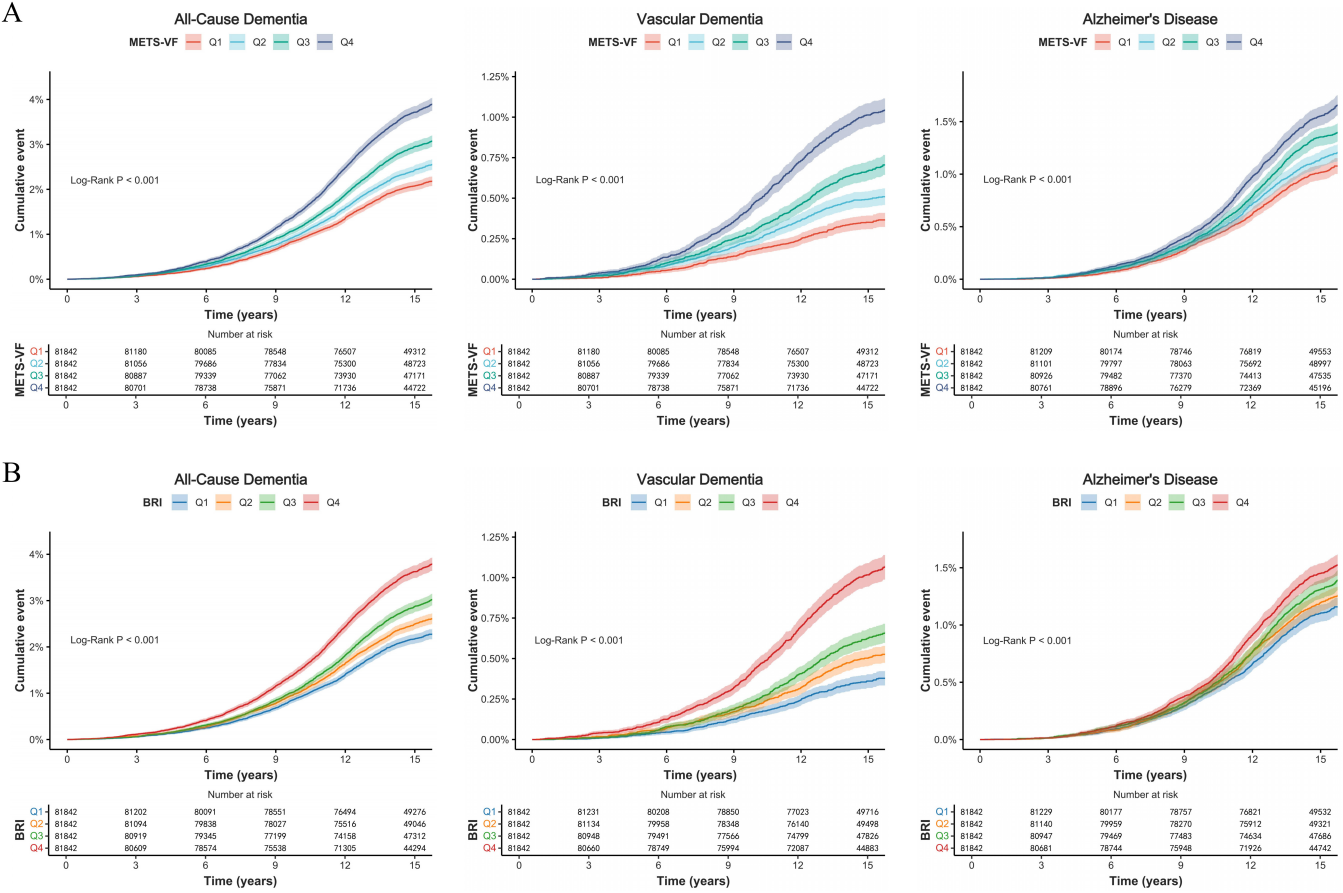
Kaplan-Meier curves for dementia outcomes according to quartiles of METS-VF and BRI. **(A)** Cumulative incidence of all-cause dementia, vascular dementia, and Alzheimer’s disease according to quartiles of METS-VF. **(B)** Cumulative incidence of all-cause dementia, vascular dementia, and Alzheimer’s disease according to quartiles of BRI. Shaded areas indicate 95% confidence intervals, and numbers at risk are shown below each curve. Log-rank *P* values are shown in the panels.

Kaplan-Meier curves (Figure 3) demonstrated significantly higher cumulative incidences of all dementia outcomes among participants in the highest quartiles of METS-VF and BRI (Q4) compared with those in the lowest quartiles (Q1) (all log-rank *P* < 0.001). In Cox proportional hazards models, METS-VF showed robust associations with increased risks of all-cause dementia, vascular dementia, and AD. Each 1-SD increase in METS-VF was associated with a 25% higher risk of all-cause dementia (HR = 1.25, 95% CI: 1.22–1.28), a 49% higher risk of vascular dementia (HR = 1.49, 95% CI: 1.41–1.58), and an 18% higher risk of AD (HR = 1.18, 95% CI: 1.13–1.22). Quartile analyses also revealed a clear dose-response relationship across all outcomes (Table 2).

**TABLE 2:** Associations between METS-VF quartiles and the risk of all-cause dementia, vascular dementia, and Alzheimer’s disease.

Variables	Overall	Quartiles of METS-VF				
		Q1 ( ≤ 6.38)	Q2 (6.38–6.80)	Q3 (6.80–7.16)	Q4 ( > 7.16)	*P for trend*
All-cause dementia						
Number of incidence	N = 8,768	N = 1,669	N = 1,938	N = 2,303	N = 2,858	
Model 1 HR (95% CI) *P*-value	1.27(1.24, 1.30) <0.001	Ref (1.0)	1.18(1.10,1.26) <0.001	1.42(1.33, 1.51) <0.001	1.80(1.70, 1.92) <0.001	< 0.001
Model 2 HR (95% CI) *P*-value	1.21(1.18, 1.24) <0.001	Ref (1.0)	1.06(0.99, 1.13) 0.085	1.23(1.15, 1.31) <0.001	1.57(1.48, 1.67) <0.001	< 0.001
Model 3 HR (95% CI) *P*-value	1.25(1.22, 1.28) <0.001	Ref (1.0)	1.10(1.03, 1.17) 0.007	1.29(1.21, 1.38) <0.001	1.70(1.59, 1.81) <0.001	< 0.001
Vascular dementia						
Number of incidence	N = 1,956	N = 282	N = 390	N = 522	N = 762	
Model 1 HR (95% CI) *P*-value	1.53(1.45, 1.60) <0.001	Ref (1.0)	1.40(1.20, 1.63) <0.001	1.90(1.64, 2.19) <0.001	2.84(2.47, 3.25) <0.001	< 0.001
Model 2 HR (95% CI) *P*-value	1.47(1.39, 1.55) <0.001	Ref (1.0)	1.18(1.02, 1.38) 0.032	1.51(1.31, 1.75) <0.001	2.40(2.09, 2.75) <0.001	< 0.001
Model 3 HR (95% CI) *P*-value	1.49(1.41, 1.58) <0.001	Ref (1.0)	1.21(1.03, 1.41) 0.018	1.55(1.34, 1.80) <0.001	2.46(2.12, 2.84) <0.001	< 0.001
Alzheimer’s disease						
Number of incidence	N = 3,951	N = 810	N = 910	N = 1,043	N = 1,188	
Model 1 HR (95% CI) *P*-value	1.20(1.16, 1.24) <0.001	Ref (1.0)	1.14(1.03, 1.25) 0.008	1.33(1.21, 1.45) <0.001	1.54(1.41, 1.69) <0.001	< 0.001
Model 2 HR (95% CI) *P*-value	1.13(1.09, 1.17) <0.001	Ref (1.0)	1.06(0.97, 1.17) 0.219	1.18(1.08, 1.30) <0.001	1.33(1.22, 1.46) <0.001	< 0.001
Model 3 HR (95% CI) *P*-value	1.18(1.13, 1.22) <0.001	Ref (1.0)	1.10(1.00, 1.22) 0.046	1.26(1.15, 1.39) <0.001	1.46(1.33, 1.61) <0.001	< 0.001

Model 1, Unadjusted; Model 2, Adjusted for age, sex, ethnicity, TDI, educational level, smoking status, alcohol consumption; Model 3, Included all variables from Model 2, and further dietary pattern, physical activity level, sleep duration, UA, eGFR, insulin use, aspirin use, antihypertensive drug use, and cholesterol-lowering medications. Ref, reference; UA, uric acid; eGFR, estimated glomerular filtration rate; TDI, Townsend deprivation index.

BRI showed similar associations for all-cause and vascular dementia. Each 1-SD increase in BRI was associated with a 16% higher risk of all-cause dementia (HR = 1.16, 95% CI: 1.14–1.19) and a 29% higher risk of vascular dementia (HR = 1.29, 95% CI: 1.24–1.35), and a 9% higher risk of AD (HR = 1.09, 95% CI: 1.05–1.12) (Table 3).

**TABLE 3:** Associations between BRI quartiles and the risk of all-cause dementia, vascular dementia, and Alzheimer’s disease.

Variables	Overall	Quartiles of BRI				
		Q1 ( ≤ 3.18)	Q2 (3.18–4.07)	Q3 (4.07–5.12)	Q4 ( > 5.12)	*P for trend*
All-cause dementia						
Number of incidence	N = 8,768	N = 1,747	N = 1,988	N = 2,260	N = 2,773	
Model 1 HR (95% CI) *P*-value	1.22(1.20, 1.24) <0.001	Ref (1.0)	1.15(1.08,1.23) <0.001	1.33(1.25, 1.41) <0.001	1.68(1.58, 1.78) <0.001	< 0.001
Model 2 HR (95% CI) *P*-value	1.14(1.11, 1.16) <0.001	Ref (1.0)	1.04(0.98, 1.11) 0.218	1.13(1.06, 1.20) <0.001	1.35(1.27, 1.43) <0.001	< 0.001
Model 3 HR (95% CI) *P*-value	1.16(1.14, 1.19) <0.001	Ref (1.0)	1.07(1.00, 1.14) 0.042	1.18(1.10, 1.26) < 0.001	1.43(1.34, 1.53) < 0.001	< 0.001
Vascular dementia						
Number of incidence	N = 1,956	N = 292	N = 401	N = 490	N = 773	
Model 1 HR (95% CI) P-value	1.40(1.35, 1.45) <0.001	Ref (1.0)	1.39(1.19, 1.61) <0.001	1.72(1.49, 1.99) <0.001	2.79(2.44, 3.20) <0.001	< 0.001
Model 2 HR (95% CI) P-value	1.29(1.24, 1.34) <0.001	Ref (1.0)	1.18(1.01, 1.38) 0.034	1.32(1.14, 1.54) <0.001	2.01(1.75, 2.31) <0.001	< 0.001
Model 3 HR (95% CI) P-value	1.29(1.24, 1.35) <0.001	Ref (1.0)	1.19(1.02, 1.39) 0.026	1.34(1.15, 1.55) <0.001	2.03(1.75, 2.35) <0.001	< 0.001
Alzheimer’s disease						
Number of incidence	N = 3,951	N = 876	N = 950	N = 1,026	N = 1,099	
Model 1 HR (95% CI) P-value	1.12(1.09, 1.16) <0.001	Ref (1.0)	1.10(1.00, 1.20) 0.052	1.20(1.10, 1.32) <0.001	1.33(1.21, 1.45) <0.001	< 0.001
Model 2 HR (95% CI) P-value	1.07(1.04, 1.10) <0.001	Ref (1.0)	1.05(0.95, 1.15) 0.342	1.10(1.00, 1.21) 0.045	1.16(1.06, 1.27) 0.002	0.008
Model 3 HR (95% CI) P-value	1.09(1.05, 1.12) <0.001	Ref (1.0)	1.06(0.97, 1.17) 0.196	1.13(1.03, 1.25) 0.012	1.21(1.10, 1.33) <0.001	< 0.001

Model 1, Unadjusted; Model 2, Adjusted for age, sex, ethnicity, TDI, educational level, smoking status, alcohol consumption; Model 3, Included all variables from Model 2, and further dietary pattern, physical activity level, sleep duration, UA, eGFR, insulin use, aspirin use, antihypertensive drug use, and cholesterol-lowering medications. Ref, reference; UA, uric acid; eGFR, estimated glomerular filtration rate; TDI, Townsend deprivation index.

**FIGURE 4:**
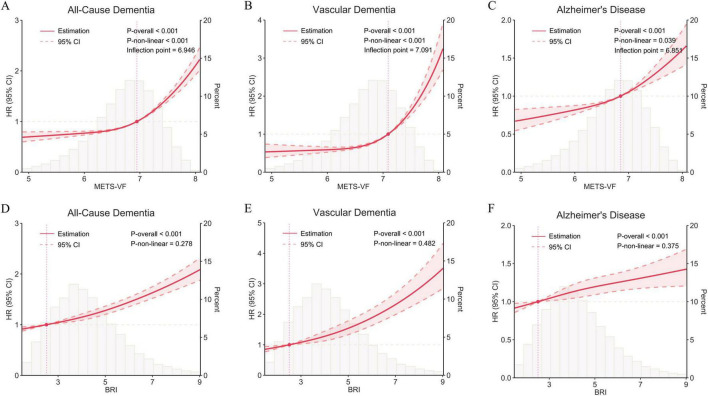
Nonlinear associations between visceral obesity indices and dementia outcomes. **(A,C,E)** Restricted cubic spline analyses of METS-VF for all-cause dementia, vascular dementia, and Alzheimer’s disease, respectively. **(B,D,F)** Restricted cubic spline analyses of BRI for all-cause dementia, vascular dementia, and Alzheimer’s disease, respectively. Solid lines indicate hazard ratios, shaded areas indicate 95% confidence intervals, and histograms show the distribution of METS-VF and BRI. Vertical dashed lines indicate threshold values for METS-VF. All models were adjusted for age, sex, ethnicity, TDI, educational level, smoking status, alcohol consumption, dietary pattern, physical activity level, sleep duration, UA, eGFR, insulin use, aspirin use, antihypertensive drug use, and cholesterol-lowering medications. *P* values for overall and non-linear associations are shown in the panels.

RCS analyses further characterized these associations (Figure 4 and Table 4). For METS-VF, significant overall associations were observed for all three outcomes (all P-overall < 0.001), and the associations were non-linear for all-cause dementia (P-non-linear < 0.001), vascular dementia (P-non-linear < 0.001), and AD (P-non-linear = 0.039), with thresholds at 6.95 (95% CI: 6.65–7.24), 7.09 (95% CI: 6.80–7.39), and 6.85 (95% CI: 6.56–7.15), respectively. These threshold values represent data-driven inflection points identified from the fitted spline curves, beyond which the association between METS-VF and dementia outcomes became steeper. Below these thresholds, each 1-unit increase in METS-VF was associated with HRs of 1.20 (95% CI: 1.13–1.27) for all-cause dementia, 1.52 (95% CI: 1.34–1.73) for vascular dementia, and 1.19 (95% CI: 1.09–1.31) for AD. Above the thresholds, the corresponding HRs were 2.07 (95% CI: 1.88–2.28), 3.34 (95% CI: 2.63–4.24), and 1.51 (95% CI: 1.32–1.72), respectively. Among participants with METS-VF values above the corresponding thresholds, 4,249 of 129,094 (3.29%) developed all-cause dementia, 867 of 96,155 (0.90%) developed vascular dementia, and 2,071 of 151,535 (1.37%) developed AD. In contrast, although BRI was also significantly associated with higher risks of all three dementia outcomes (all P-overall < 0.001), there was no evidence of non-linearity for all-cause dementia (P-non-linear = 0.278), vascular dementia (P-non-linear = 0.482), or AD (P-non-linear = 0.375), indicating approximately linear associations.

**TABLE 4:** Threshold effect analyses of METS-VF on incident all-cause dementia, vascular dementia, and Alzheimer’s disease.

Variables	Incident cases, n/N (%)	HR (95% CI)	*P*-value
All-cause dementia			
Fitting by 2-piecewise Cox proportional hazards model			
Linear effect		1.44 (1.38–1.50)	< 0.001
Threshold value		6.95 (6.65–7.24)	
METS-VF < 6.95	4,519/198,274 (2.28)	1.20 (1.13–1.27)	< 0.001
METS-VF ≥ 6.95	4,249/129,094 (3.29)	2.07 (1.88–2.28)	< 0.001
*P* for log-likelihood ratio test			< 0.001
Vascular dementia			
Fitting by 2-piecewise Cox proportional hazards model			
Linear effect		1.91 (1.74–2.10)	< 0.001
Threshold value		7.09 (6.80–7.39)	
METS-VF < 7.09	1,089/231,213 (0.47)	1.52 (1.34–1.73)	< 0.001
METS-VF ≥ 7.09	867/96,155 (0.90)	3.34 (2.63–4.24)	< 0.001
*P* for log-likelihood ratio test			< 0.001
Alzheimer’s disease			
Fitting by 2-piecewise Cox proportional hazards model			
Linear effect		1.30 (1.23–1.38)	< 0.001
Threshold value		6.85 (6.56–7.15)	
METS-VF < 6.85	1,880/175,833 (1.07)	1.19 (1.09–1.31)	< 0.001
METS-VF ≥ 6.85	2,071/151,535 (1.37)	1.51 (1.32–1.72)	< 0.001
*P* for log-likelihood ratio test			0.017

Threshold effect analyses were performed to evaluate the nonlinear associations of METS-VF with incident all-cause dementia, vascular dementia, and Alzheimer’s disease. When evidence of nonlinearity was observed, a 2-piecewise Cox proportional hazards regression model was fitted to estimate the associations below and above the threshold value. Threshold values are presented with 95% CIs in parentheses. The “Linear effect” represents the overall association estimated from the single-line Cox model, whereas the estimates for METS-VF < threshold and METS-VF ≥ threshold were derived from the 2-piecewise Cox proportional hazards model. All models were adjusted for age, sex, ethnicity, Townsend Deprivation Index, educational level, smoking status, alcohol consumption, dietary pattern, physical activity level, sleep duration, uric acid, estimated glomerular filtration rate, insulin use, aspirin use, antihypertensive drug use, and cholesterol-lowering medications. CI, confidence interval; HR, hazard ratio; METS-VF, metabolic score for visceral fat; W, threshold value.

In addition, no evidence of multicollinearity was observed in the fully adjusted model (Model 3) when METS-VF and BRI were entered separately, as all generalized variance inflation factor (GVIF) values were < 2 (Supplementary Tables S2, S3).

### 3.4 Visceral obesity and genetic susceptibility for AD

**FIGURE 5:**
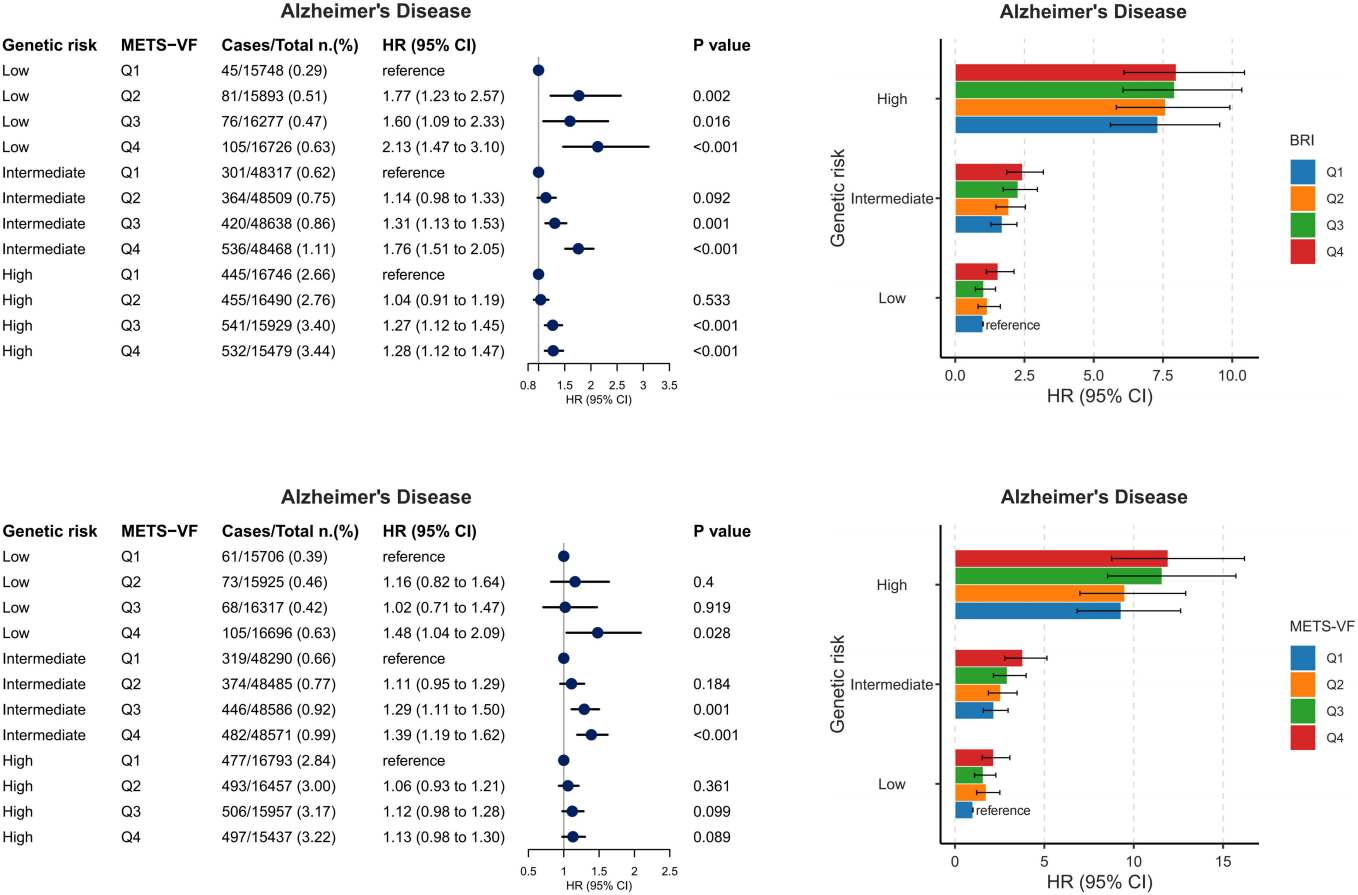
Joint associations of visceral obesity indices and PRS with Alzheimer’s disease. Associations of BRI and METS-VF quartiles with Alzheimer’s disease across low, intermediate, and high genetic risk groups. Cases/total, hazard ratios with 95% confidence intervals, and *P* values are shown in the figure. Quartile 1 served as the reference. All models were adjusted for age, sex, ethnicity, TDI, educational level, smoking status, alcohol consumption, dietary pattern, physical activity level, sleep duration, UA, eGFR, insulin use, aspirin use, antihypertensive drug use, and cholesterol-lowering medications.

In analyses of ALZPRS (*n* = 323,048), the associations of BRI and METS-VF quartiles with incident AD stratified by genetic risk are shown in Figure 5. For BRI, compared with Q1, the risk of AD increased significantly across higher quartiles among participants with low genetic risk, with HRs of 1.77 (95% CI, 1.23–2.57; *P* = 0.002), 1.60 (95% CI, 1.09–2.33; *P* = 0.016), and 2.13 (95% CI, 1.47–3.10; *P* < 0.001) for Q2, Q3, and Q4, respectively. Among those with intermediate genetic risk, the corresponding HRs were 1.14 (95% CI, 0.98–1.33; *P* = 0.092), 1.31 (95% CI, 1.13–1.53; *P* = 0.001), and 1.76 (95% CI, 1.51–2.05; *P* < 0.001). In the high genetic risk group, the associations were attenuated, with HRs of 1.04 (95% CI, 0.91–1.19; *P* = 0.533), 1.27 (95% CI, 1.12–1.45; *P* < 0.001), and 1.28 (95% CI, 1.12–1.47; *P* < 0.001) for Q2, Q3, and Q4, respectively.

A similar pattern was observed for METS-VF. In the low genetic risk group, Q4 was significantly associated with a higher risk of AD (HR, 1.48; 95% CI, 1.04–2.09; *P* = 0.028), whereas Q2 and Q3 were not significant. In the intermediate genetic risk group, the HRs were 1.11 (95% CI, 0.95–1.29; *P* = 0.184), 1.29 (95% CI, 1.11–1.50; *P* = 0.001), and 1.39 (95% CI, 1.19–1.62; P < 0.001) for Q2, Q3, and Q4, respectively. In the high genetic risk group, no significant associations were observed, with HRs of 1.06 (95% CI, 0.93–1.21; *P* = 0.361), 1.12 (95% CI, 0.98–1.28; *P* = 0.099), and 1.13 (95% CI, 0.98–1.30; *P* = 0.089) for Q2, Q3, and Q4, respectively. Overall, these findings suggest that the associations of BRI and METS-VF with AD were more evident among participants with low or intermediate genetic risk than among those with high genetic risk.

To extend the stratified findings, interaction tests between METS-VF or BRI and PRS were performed on additive and multiplicative scales. Synergistic additive interactions and antagonistic interactions on the multiplicative scale were observed (Supplementary Table S4).

### 3.5 Metabolic mediation analyses

**FIGURE 6:**
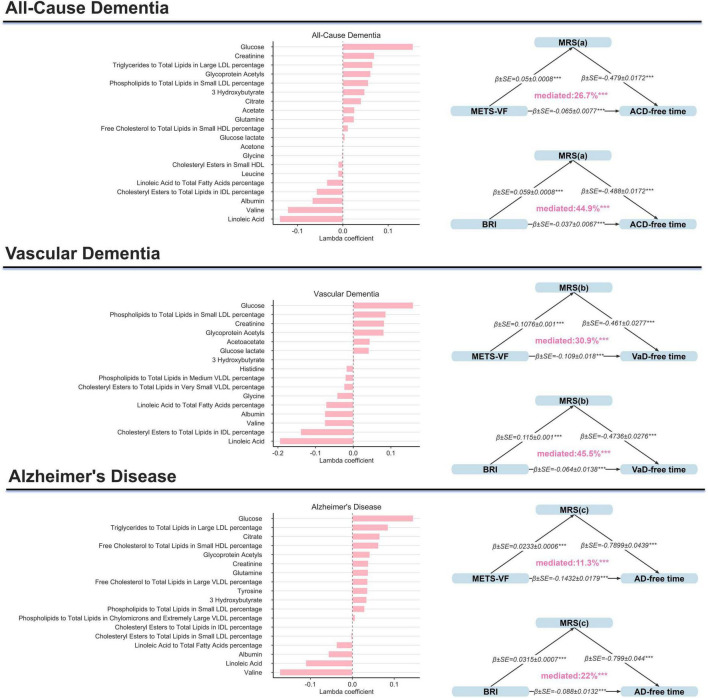
Mediation of metabolic risk scores in associations between visceral obesity and dementia-free survival time. Schematic representation of mediation analyses assessing indirect effects of metabolic risk scores on the associations of METS-VF and BRI with dementia-free survival time. Metabolic risk scores were derived from LASSO-Cox selected metabolites. All models were adjusted for age, sex, ethnicity, TDI, educational level, smoking status, alcohol consumption, dietary pattern, physical activity level, sleep duration, UA, eGFR, insulin use, aspirin use, antihypertensive drug use, and cholesterol-lowering medications. Mediation proportions indicate the percentage of total associations statistically explained by metabolic dysfunction.

To investigate potential metabolic pathways linking METS-VF and BRI with dementia risk, metabolomic analyses were performed in participants with available metabolomic data (*n* = 184,183). LASSO-Cox regression identified key metabolic biomarkers associated with all-cause dementia, vascular dementia, and AD (Supplementary Figure S1). These biomarkers were used to construct metabolic risk scores, which were incorporated into mediation models examining dementia-free survival time (Figure 6).

Mediation analysis showed that the MRS mediated 26.7 and 44.9% of the associations of METS-VF and BRI with all-cause dementia, respectively; 30.9 and 45.5% of the associations with vascular dementia; and 11.3 and 22.0% of the associations with AD. These results suggest that metabolic perturbations may partly explain the observed associations of METS-VF and BRI with dementia-related outcomes.

### 3.6 Subgroup and sensitivity analyses

Subgroup analyses showed that higher METS-VF and BRI were associated with increased dementia risk across most strata. Significant interaction by age was observed for both METS-VF and BRI (both *P* for interaction < 0.001), with stronger associations in participants aged < 60 years than in those aged ≥ 60 years. A significant interaction with sleep duration was observed for BRI (*P* for interaction = 0.030), whereas the interaction for METS-VF was not statistically significant (*P* for interaction = 0.082). No significant interactions were found for sex, ethnicity, educational level, smoking status, alcohol consumption, dietary pattern, or physical activity (all *P* for interaction > 0.05) (Supplementary Table S5).

Sensitivity analyses yielded results broadly consistent with the primary analyses. Of the incident cases, 683 of 8,768 (7.79%) all-cause dementia cases, 202 of 1,956 (10.33%) vascular dementia cases, and 286 of 3,951 (7.24%) AD cases were diagnosed within the first 5 years of follow-up. Excluding these early-incident cases did not materially alter the associations of METS-VF and BRI with all three dementia outcomes. Similar findings were observed when all-cause mortality was treated as a competing event and after excluding participants with underweight at baseline (Supplementary Tables S6–S10). Likewise, in the trajectory analyses, further exclusion of incident dementia cases occurring within 5 years after the second follow-up did not materially alter the observed associations. Overall, these findings support the robustness of the main results (Supplementary Tables S11, S12).

### 3.7 Incremental explanatory contribution of METS-VF and BRI beyond BMI and WC

To further assess whether METS-VF and BRI provided explanatory value beyond traditional obesity indices, we performed a two-step residual-based analysis. Beyond the dementia risk explained by BMI alone, both METS-VF and BRI remained significantly associated with higher dementia risk and explained additional variation, with ΔR^2^ values of 0.34 and 0.36%, respectively. Beyond the risk explained by WC alone, the incremental explanatory contribution was greater, with ΔR^2^ values of 1.33% for METS-VF and 1.61% for BRI. In the secondary models, both METS-VF and BRI were independently associated with higher dementia risk (all *P* < 0.001) (Supplementary Table S13). These findings indicate that METS-VF and BRI provide explanatory value beyond that captured by BMI or WC alone, rather than simply serving as proxies for conventional obesity measures.

### 3.8 Changes in anthropometric and metabolic markers before dementia diagnosis

Among participants who subsequently developed dementia, significant longitudinal changes were observed in several anthropometric and metabolic markers between baseline and the first follow-up visit. WC showed a significant upward shift despite an unchanged median, increasing from 92 (80, 100) to 92 (84, 102) (*Z* = 5.52, *P* < 0.001). FBG also increased significantly, from 4.91 (4.56, 5.38) to 5.07 (4.76, 5.47) (*Z* = 3.46, *P* = 0.001). In contrast, TG showed a modest but significant decrease, from 1.44 (1.04, 2.05) to 1.39 (0.97, 1.94) (*Z* = 2.03, *P* = 0.042), whereas HDL-C increased significantly, from 1.38 (1.12, 1.72) to 1.48 (1.17, 1.79) (*Z* = 4.17, *P* < 0.001). No significant change was observed for BMI, which remained at 26.42 at both time points (*Z* = 0.83, *P* = 0.408) (Supplementary Tables S14, S15). These findings suggest that, before dementia diagnosis, individuals may experience progressive central adiposity and metabolic alterations despite relatively stable overall body mass.

## 4 Discussion

In this large prospective cohort study, we found three main results. First, among the tested indices, METS-VF and BRI showed the strongest correlations with imaging-derived visceral adiposity and better predictive performance for dementia outcomes than conventional obesity measures. Second, higher METS-VF and BRI were associated with increased risks of all-cause dementia, vascular dementia, and AD, although the association between BRI and AD was attenuated after full adjustment but remained statistically significant. Third, these associations were supported by subgroup, sensitivity, and additional analyses, including metabolomic mediation, incremental explanatory analyses beyond BMI and WC, and longitudinal changes in anthropometric and metabolic markers before dementia diagnosis.

A key implication of our findings is that dementia-related obesity risk may depend less on body size itself than on metabolically adverse fat distribution. VAT is more strongly related than subcutaneous adiposity to FBG, TG, low HDL-C, hypertension, diabetes, and metabolic syndrome, suggesting that visceral adiposity may be more closely linked to vascular and neurodegenerative vulnerability (13). This framework also aligns with current dementia-prevention literature, which increasingly emphasizes the contribution of modifiable cardiometabolic risk factors to later cognitive decline (34). Our results therefore support the view that visceral obesity indices may be more informative than BMI-based definitions of obesity when the outcome of interest is dementia risk. To enhance understanding of the proposed pathway linking visceral obesity, metabolic dysfunction, and dementia risk, we have included an integrative schematic in Supplementary Figure S2.

This interpretation may help explain the inconsistency of the obesity-dementia literature, much of which has relied on BMI. BMI does not distinguish visceral from subcutaneous fat and is particularly vulnerable to reverse causation in dementia research, because body composition can change during the prodromal phase before clinical diagnosis. Recent studies have highlighted this heterogeneity. For example, a 2025 review and meta-analysis with Mendelian-randomization analyses reported that central or visceral obesity was not consistently associated with cognitive impairment or dementia, while some general obesity measures even showed inverse associations in older populations (35). Such discrepant findings may reflect differences in age at exposure assessment, outcome definitions, and the limitations of conventional anthropometric measures in later life. In this context, our residual-based analyses showing additional explanatory value of METS-VF and BRI beyond BMI and WC are important, because they suggest that these indices capture risk information not fully represented by traditional obesity measures.

The stronger associations observed for vascular dementia are biologically plausible. Visceral adiposity is tightly linked to insulin resistance, dyslipidemia, endothelial dysfunction, chronic low-grade inflammation, and vascular injury, all of which have been implicated in cerebral small-vessel disease and impaired neurovascular regulation. By contrast, the association with AD was more modest, particularly for BRI after full adjustment. One explanation is that METS-VF may better capture the metabolic consequences of visceral adiposity because it incorporates TG and HDL-C, whereas BRI is primarily a body-shape-based index. However, an alternative explanation should also be considered. Because TG and HDL-C may lie on the causal pathway linking visceral adiposity to dementia, adjustment for these factors could preferentially attenuate the association for BRI and thereby constitute partial over-adjustment. In contrast, because these metabolic components are embedded within METS-VF, the retained association for METS-VF may indicate that this index better captures the metabolically relevant component of visceral obesity rather than simply being less confounded. Thus, the attenuation of BRI after full adjustment should not necessarily be interpreted as evidence of inferior biological relevance, but may instead reflect differences in construct composition and possible adjustment for mediators rather than confounders (17).

The threshold pattern observed for METS-VF also deserves clinical interpretation. In our cohort, 3.29, 0.90, and 1.37% of participants had METS-VF values above the corresponding estimated risk-based inflection points for all-cause dementia, vascular dementia, and AD, respectively, indicating that these inflection points were not driven solely by a handful of extreme values. However, these spline-derived values should not be regarded as established clinical cut-offs, diagnostic thresholds, or treatment targets for visceral obesity. Within this study, they reflect risk-based inflection points indicating a statistically detectable change in the strength of the association, rather than clinically meaningful or actionable thresholds. METS-VF is a composite index derived from anthropometric and metabolic components and was developed as a surrogate estimator of visceral fat rather than an imaging-based classification tool (17). By comparison, direct quantification of VAT still depends mainly on imaging methods such as CT and MRI (15). Thus, the present thresholds are more appropriately interpreted as risk-based inflection points within this cohort, beyond which the association between visceral adiposity-related metabolic burden and dementia risk becomes steeper. They may therefore complement, rather than replace, conventional approaches to assessing central obesity and visceral adiposity in clinical and epidemiological settings (7, 24).

Our metabolomic mediation analyses provide hypothesis-generating evidence that downstream metabolic dysfunction may partly account for the observed associations between visceral obesity indices and dementia-related outcomes. The observed mediation proportions, particularly for all-cause dementia and vascular dementia, suggest that glucose- and lipid-related metabolic perturbations may contribute to these associations. However, these findings should not be interpreted as evidence of a causal mediation pathway. Because visceral obesity indices and metabolomic biomarkers were measured contemporaneously, the temporal ordering between exposure and mediator could not be established. In addition, residual confounding and reverse causation cannot be fully excluded. Therefore, the mediation results should be viewed as exploratory evidence supporting a potential metabolic link between visceral obesity indices and dementia risk, rather than as definitive proof of mechanism. This interpretation is consistent with broader evidence that cardiometabolic dysfunction is associated with dementia onset and progression, including reports linking type 2 diabetes, hypertension, and hyperlipidemia with progression from mild cognitive impairment to dementia, particularly AD (36).

The neurobiological plausibility of our findings is also supported by recent neuroimaging and cognitive studies. In the UK Biobank, higher BMI was associated with lower gray matter volume, greater white-matter hyperintensity burden, and poorer fluid intelligence (37). In addition, a recent population-based study showed that higher BRI was associated with worse cognitive performance in older adults (21). Together, these findings suggest that obesity-related brain vulnerability may be detectable at the level of brain structure and cognition before the occurrence of clinically diagnosed dementia, and that indices capturing metabolically adverse adiposity may be more informative than BMI alone.

Our additional longitudinal analysis among participants who subsequently developed dementia provides further insight into potential prediagnostic changes. We observed that waist circumference and fasting blood glucose increased before diagnosis, whereas BMI remained relatively stable. At first glance, this pattern may appear different from the commonly reported prediagnostic decline in body weight or BMI in AD and related dementias. However, these findings are not necessarily contradictory, because different anthropometric and metabolic markers may capture distinct aspects of the prodromal process. Previous studies have shown that body weight and BMI may decline before AD diagnosis, potentially related to neurodegeneration in appetite- and energy-regulating brain regions, which may also contribute to reverse causation in the obesity–dementia literature (8, 9, 38). In addition, higher AD genetic risk has been associated with lower late-life BMI, further supporting the possibility that lower body mass in later life may partly reflect preclinical disease processes rather than a truly protective effect (39). At the same time, recent longitudinal evidence suggests that cardiometabolic trajectories preceding dementia may be heterogeneous across markers: for example, declines in BMI and waist circumference have been reported in some cohorts, whereas trajectories of glucose and other metabolic factors may show less consistent patterns (40). Recent neuroimaging evidence further suggests that the relationship between adiposity and dementia-related brain vulnerability is complex and may vary by age and sex (36). In our cohort, central adiposity and glycemic dysregulation appeared to worsen before diagnosis even though BMI remained relatively stable, further highlighting the limitation of BMI as a sole marker of dementia-related metabolic risk. This pattern also provides some support for the rationale for indices such as METS-VF and BRI, which may better capture adverse body-fat distribution and metabolic burden than conventional anthropometric measures alone.

Consistent with this, stratified analyses by polygenic risk score (PRS) further suggested that the associations between visceral obesity indices and dementia outcomes were more pronounced among individuals with low to intermediate genetic risk. Several non-mutually exclusive explanations may be considered. First, a potential ceiling effect in individuals at high genetic risk may attenuate the relative contribution of modifiable metabolic factors. Second, genetic susceptibility may reflect the influence of non-modifiable biological pathways that are more dominant in high-PRS groups, whereas environmental and metabolic factors, including visceral adiposity, may be more relevant in those with lower genetic risk. Third, individuals with lower genetic predisposition may be more sensitive to adverse metabolic exposures, resulting in stronger relative associations. However, these interpretations remain speculative. The interaction analyses were exploratory and may be underpowered to detect effect modification across genetic risk strata; therefore, these findings should be interpreted with caution and warrant replication in independent cohorts.

Several aspects of our analyses strengthen the robustness of these findings. The associations remained materially unchanged after accounting for all-cause mortality as a competing event, after excluding incident dementia cases occurring within the first 5 years of follow-up, and after excluding participants with underweight at baseline. In the trajectory analyses, additional exclusion of cases occurring within 5 years after the second follow-up also did not materially alter the observed patterns. Moreover, no evidence of problematic multicollinearity was observed when METS-VF and BRI were entered separately in the fully adjusted models. Together, these analyses reduce concerns that the main findings were driven solely by reverse causation, competing risk, or model instability.

Overall, these findings suggest that indices reflecting metabolically adverse visceral adiposity may be more strongly associated with dementia risk than general obesity measures, and that METS-VF may provide additional risk-related information beyond conventional anthropometric measures. These findings may be particularly relevant for epidemiological research and population-level risk stratification; however, given the observational design and the surrogate nature of these indices, further validation is needed before clinical implementation.

## 5 Strengths and limitations

This study has several notable strengths. It was conducted in a large prospective cohort with long-term follow-up and sufficient numbers of incident all-cause dementia, vascular dementia, and Alzheimer’s disease, enabling subtype-specific analyses. We systematically compared several visceral obesity indices and validated their performance against imaging-derived visceral adiposity, which strengthened the rationale for focusing on METS-VF and BRI. We also incorporated multiple complementary analytical approaches, including dose-response analyses, subgroup and sensitivity analyses, metabolomic mediation, genetic stratification, trajectory analyses, and incremental explanatory analyses beyond conventional obesity measures. Together, these analyses provided a more comprehensive evaluation of the relationship between visceral obesity and dementia risk than would be possible with BMI alone.

This study also has limitations. The observational design precludes firm causal inference, and unmeasured or residual confounding cannot be excluded. Because the UK Biobank is not fully representative of the general population, the external generalizability of the findings may be limited. Dementia has a long preclinical phase, and although we undertook several analyses to reduce reverse-causation-related bias, such bias cannot be ruled out entirely. In addition, the obesity indices evaluated here are derived proxies rather than direct measures of visceral adiposity, and repeated anthropometric and metabolic assessments were only available in a subset of participants. Finally, the mediation analyses should be interpreted cautiously and regarded as hypothesis-generating. Although these analyses may provide insight into potential metabolic links between visceral obesity indices and dementia risk, mediation does not imply causality. Because exposure and mediator were measured contemporaneously, temporal precedence could not be established, and residual confounding and reverse causation cannot be fully excluded.

## 6 Conclusion

Visceral obesity, particularly as captured by METS-VF, was independently associated with increased risks of all-cause dementia, vascular dementia, and Alzheimer’s disease. Compared with conventional obesity measures, visceral obesity-related indices appeared to provide more informative characterization of dementia risk. These findings support moving beyond BMI-centric approaches in dementia research and highlight visceral adiposity-related metabolic dysfunction as a potential focus for future risk stratification and prevention-oriented studies.

## Data Availability

The datasets analyzed in this study are subject to access restrictions imposed by the UK Biobank. Data are available upon successful application through the UK Biobank Access Management System and cannot be shared publicly by the authors. Requests to access these datasets should be directed to UK Biobank Access Management Team via the following website: https://www.ukbiobank.ac.uk/enable-your-research/apply-for-access.
