# Supplementary material for: Associations of novel visceral obesity indices (METS-VF and BRI) with dementia: the role of metabolic mediators and genetic susceptibility
Source: Front Nutr. 2026 May 18;13:1800772. doi: 10.3389/fnut.2026.1800772 (PMC13222951; doi:10.3389/fnut.2026.1800772)
Supplement: Supplementary file 1 [file Data_Sheet_1.docx]

**Associations of Novel Visceral Obesity Indices (METS-VF and BRI) with Dementia: The Role of Metabolic Mediators and Genetic Susceptibility**

**Table S1.** Assessment of Healthy Diet Score in the UK Biobank

| **Diet** | **UK Biobank field ID** | **Description** | **Healthy diet score** |
| --- | --- | --- | --- |
| Vegetable | 1289 | Cooked vegetable intake | 1 for ≥4 tablespoons/day |
|  | 1299 | Salad/raw vegetable intake | 0 for <4 tablespoons/day |
| Fruit | 1309 | Fresh fruit intake | 1 for ≥3 pieces/day |
|  | 1319 | Dried fruit intake | 0 for <3 pieces/day |
| Fish | 1329 | Oily fish intake | 1 for ≥2 times/week |
|  | 1339 | Non-oily fish intake | 0 for <2 times/week |
| Processed meat | 1349 | Processed meat intake | 1 for <2 times/week 0 for ≥2 times/week |
| Unprocessed red meat | 1369 | Beef intake | 1 for <2 times/week |
|  | 1379 | Lamb/mutton intake | 0 for ≥2 times/week |
|  | 1389 | Pork intake | 0 for ≥2 times/week |

**Notes:** The healthy diet score was derived from UK Biobank dietary questionnaire items. Component criteria are shown above; scores were summed across components.

**Table S2. Collinearity diagnostics for covariates including METS-VF in Model 3**

|  | GVIF | | |
| --- | --- | --- | --- |
| Variable | All-cause dementia | Vascular dementia | Alzheimer’s disease |
| Age | 1.17 | 1.15 | 1.15 |
| Sex | 1.42 | 1.39 | 1.43 |
| Ethnicity | 1.07 | 1.06 | 1.06 |
| TDI | 1.09 | 1.10 | 1.08 |
| Education | 1.05 | 1.04 | 1.04 |
| Smoking status | 1.23 | 1.14 | 1.12 |
| Alcohol status | 1.11 | 1.12 | 1.10 |
| Diet | 1.02 | 1.03 | 1.02 |
| Physical activity | 1.05 | 1.06 | 1.04 |
| Sleep duration | 1.01 | 1.01 | 1.01 |
| Cholesterol-lowering medication | 1.52 | 1.54 | 1.49 |
| Blood pressure medication | 1.39 | 1.41 | 1.37 |
| Insulin | 1.07 | 1.09 | 1.06 |
| Aspirin | 1.34 | 1.36 | 1.32 |
| UA | 1.67 | 1.60 | 1.70 |
| eGFR | 1.39 | 1.39 | 1.38 |
| METS-VF | 1.28 | 1.29 | 1.28 |

**Notes:** To avoid potential multicollinearity, METS-VF was entered separately in Model 3. Multicollinearity among variables in Model 3 was assessed using the generalized variance inflation factor (GVIF), with GVIF < 2 indicating no evidence of multicollinearity.

**Abbreviations:** eGFR, estimated glomerular filtration rate; GVIF, generalized variance inflation factor; METS-VF, metabolic score for visceral fat; TDI, Townsend deprivation index; UA, uric acid.

**Table S3. Collinearity diagnostics for covariates including BRI in Model 3**

|  | **GVIF** | | |
| --- | --- | --- | --- |
| **Variable** | **All-cause dementia** | **Vascular dementia** | **Alzheimer’s disease** |
| Age | 1.16 | 1.14 | 1.15 |
| Sex | 1.37 | 1.32 | 1.38 |
| Ethnicity | 1.07 | 1.06 | 1.06 |
| TDI | 1.09 | 1.10 | 1.09 |
| Education | 1.04 | 1.04 | 1.04 |
| Smoking status | 1.13 | 1.14 | 1.12 |
| Alcohol status | 1.11 | 1.12 | 1.10 |
| Diet | 1.02 | 1.02 | 1.02 |
| Physical activity | 1.05 | 1.06 | 1.05 |
| Sleep duration | 1.01 | 1.01 | 1.01 |
| Cholesterol-lowering medication | 1.51 | 1.53 | 1.49 |
| Blood pressure medication | 1.40 | 1.41 | 1.37 |
| Insulin | 1.08 | 1.10 | 1.07 |
| Aspirin | 1.34 | 1.36 | 1.32 |
| UA | 1.64 | 1.58 | 1.68 |
| eGFR | 1.39 | 1.39 | 1.38 |
| BRI | 1.30 | 1.29 | 1.30 |

**Notes:** To avoid potential multicollinearity, BRI was entered separately in Model 3. Multicollinearity among variables in Model 3 was assessed using the generalized variance inflation factor (GVIF), with GVIF < 2 indicating no evidence of multicollinearity.

**Abbreviations:** BRI, body roundness index; eGFR, estimated glomerular filtration rate; GVIF, generalized variance inflation factor; TDI, Townsend deprivation index; UA, uric acid.

**Table S4. Additive and multiplicative interactions between adiposity indices and Alzheimer’s disease polygenic risk score**

| **Interactive items** | **METS-VF Model 1** | **METS-VF Model 2** | **METS-VF Model 3** | **BRI Model 1** | **BRI Model 2** | **BRI Model 3** |
| --- | --- | --- | --- | --- | --- | --- |
| **Additive effects** | | | | | | |
| RERI | 0.85 (0.46–1.23) | 0.67 (0.32–1.01) | 0.75 (0.39–1.11) | 0.64 (0.31–0.96) | 0.44 (0.16–0.72) | 0.51 (0.21–0.80) |
| AP | 0.16 (0.12–0.20) | 0.13 (0.09–0.18) | 0.15 (0.11–0.19) | 0.13 (0.09–0.17) | 0.10 (0.05–0.14) | 0.11 (0.07–0.15) |
| SI | 1.25 (1.19–1.32) | 1.20 (1.14–1.27) | 1.23 (1.17–1.29) | 1.20 (1.14–1.26) | 1.14 (1.08–1.20) | 1.16 (1.10–1.22) |
| Multiplicative effect | 0.92 (0.87–0.96) | 0.92 (0.87–0.96) | 0.92 (0.87–0.96) | 0.92 (0.88–0.96) | 0.92 (0.88–0.96) | 0.92 (0.88–0.97) |

**Notes:** Model 1: Unadjusted. Model 2: Adjusted for age, sex, ethnicity, TDI, educational level, smoking status, alcohol consumption. Model 3: Included all variables from Model 2, and further dietary pattern, physical activity level, sleep duration, UA, eGFR, insulin use, aspirin use, antihypertensive drug use, and cholesterol-lowering medications.

**Abbreviations:** AP, proportion attributable to interaction; CI, confidence interval; RERI, relative excess risk due to interaction; SI, synergy index; UA, uric acid; eGFR, estimated glomerular filtration rate; TDI, Townsend deprivation index.

**Table S5. Subgroup analyses of the associations of METS-VF and BRI with the study outcome**

|  | **METS-VF** | | | | **BRI** | | | |
| --- | --- | --- | --- | --- | --- | --- | --- | --- |
| **Variables** | **HR** | **95% CI** | **P value** | **P for interaction** | **HR** | **95% CI** | **P value** | **P for interaction** |
| **Age** |  |  |  | <0.001 |  |  |  | <0.001 |
| Age < 60 y | 1.24 | (1.19, 1.29) | <0.001 |  | 1.20 | (1.16, 1.24) | <0.001 |  |
| Age ≥ 60 y | 1.04 | (1.00, 1.07) | 0.033 |  | 1.08 | (1.05, 1.12) | <0.001 |  |
| **Sex** |  |  |  | 0.231 |  |  |  | 0.935 |
| Male | 1.31 | (1.26, 1.37) | <0.001 |  | 1.18 | (1.14, 1.21) | <0.001 |  |
| Female | 1.20 | (1.16, 1.24) | <0.001 |  | 1.13 | (1.10, 1.17) | <0.001 |  |
| **Race** |  |  |  | 0.938 |  |  |  | 0.404 |
| Non-White | 1.34 | (1.17, 1.53) | <0.001 |  | 1.14 | (1.03, 1.27) | 0.009 |  |
| White | 1.25 | (1.22, 1.28) | <0.001 |  | 1.16 | (1.14, 1.19) | <0.001 |  |
| **Education** |  |  |  | 0.462 |  |  |  | 0.274 |
| With a university degree | 1.24 | (1.21, 1.28) | <0.001 |  | 1.15 | (1.12, 1.17) | <0.001 |  |
| No university degree | 1.26 | (1.20, 1.33) | <0.001 |  | 1.22 | (1.16, 1.28) | <0.001 |  |
| **Smoking** |  |  |  | 0.955 |  |  |  | 0.621 |
| Never | 1.25 | (1.21, 1.30) | <0.001 |  | 1.18 | (1.14, 1.22) | <0.001 |  |
| Previous | 1.25 | (1.20, 1.30) | <0.001 |  | 1.14 | (1.10, 1.18) | <0.001 |  |
| Current | 1.24 | (1.15, 1.34) | <0.001 |  | 1.18 | (1.11, 1.26) | <0.001 |  |
| **Alcohol** |  |  |  | 0.577 |  |  |  | 0.631 |
| Never | 1.29 | (1.18, 1.42) | 0.003 |  | 1.18 | (1.10, 1.27) | <0.001 |  |
| Previous | 1.30 | (1.18, 1.43) | <0.001 |  | 1.20 | (1.11, 1.28) | <0.001 |  |
| Current | 1.24 | (1.21, 1.28) | <0.001 |  | 1.16 | (1.13, 1.18) | <0.001 |  |
| **Diet** |  |  |  | 0.616 |  |  |  | 0.537 |
| Healthy | 1.25 | (1.20, 1.29) | <0.001 |  | 1.16 | (1.13, 1.19) | <0.001 |  |
| Unhealthy | 1.26 | (1.21, 1.28) | <0.001 |  | 1.17 | (1.13, 1.21) | <0.001 |  |
| **Physical activity** |  |  |  | 0.408 |  |  |  | 0.424 |
| Low | 1.23 | (1.14, 1.31) | <0.001 |  | 1.13 | (1.07, 1.19) | <0.001 |  |
| Moderate | 1.29 | (1.21, 1.37) | <0.001 |  | 1.17 | (1.12, 1.23) | <0.001 |  |
| High | 1.24 | (1.20, 1.28) | <0.001 |  | 1.16 | (1.13, 1.20) | <0.001 |  |
| **Sleep** |  |  |  | 0.082 |  |  |  | 0.030 |
| <7h | 1.18 | (1.13, 1.24) | <0.001 |  | 1.11 | (1.07, 1.16) | <0.001 |  |
| 7h–8h | 1.26 | (1.22, 1.30) | <0.001 |  | 1.18 | (1.14, 1.21) | <0.001 |  |
| >8h | 1.28 | (1.18, 1.38) | <0.001 |  | 1.16 | (1.10, 1.23) | <0.001 |  |

**Notes:** Subgroup analyses were conducted using the fully adjusted Cox proportional hazards model (Model 3). Hazard ratios (HRs) and 95% confidence intervals (CIs) are presented for each 1-standard deviation (SD) increase in METS-VF and BRI. METS-VF and BRI were entered separately in the models. *P* for interaction was calculated to assess potential effect modification across strata of age, sex, race, education, smoking status, alcohol consumption, dietary pattern, physical activity, and sleep duration.

**Abbreviations:** BRI, body roundness index; CI, confidence interval; HR, hazard ratio; METS-VF, metabolic score for visceral fat; SD, standard deviation.

**Table S6. Results of multivariate competing risk models for METS-VF and BRI quartiles**

|  |  | **All-cause dementia** | | **Vascular dementia** | | **Alzheimer’s disease** | |
| --- | --- | --- | --- | --- | --- | --- | --- |
| **Indicator** | **Quartiles** | **HR (95% CI)** | **P value** | **HR (95% CI)** | **P value** | **HR (95% CI)** | **P value** |
| **METS-VF** | Q1 (≤6.38) | Ref |  | Ref |  | Ref |  |
|  | Q2 (6.38–6.80) | 1.12 (1.05–1.20) | 0.001 | 1.26 (1.07–1.48) | 0.006 | 1.12 (1.01–1.24) | 0.026 |
|  | Q3 (6.80–7.16) | 1.30 (1.22–1.40) | <0.001 | 1.57 (1.34–1.84) | <0.001 | 1.24 (1.12–1.37) | <0.001 |
|  | Q4 (>7.16) | 1.60 (1.49–1.72) | <0.001 | 2.28 (1.95–2.67) | <0.001 | 1.40 (1.26–1.55) | <0.001 |
|  | Increase per 1-SD | 1.24 (1.21–1.27) | <0.001 | 1.47 (1.38–1.57) | <0.001 | 1.17 (1.13–1.21) | <0.001 |
| **BRI** | Q1 (≤3.18) | Ref |  | Ref |  | Ref |  |
|  | Q2 (3.18–4.07) | 1.08 (1.01–1.16) | 0.032 | 1.21 (1.03–1.42) | 0.022 | 1.06 (0.96–1.17) | 0.252 |
|  | Q3 (4.07–5.12) | 1.18 (1.09–1.26) | <0.001 | 1.38 (1.17–1.64) | <0.001 | 1.12 (1.01–1.25) | 0.026 |
|  | Q4 (>5.12) | 1.37 (1.28–1.47) | <0.001 | 1.89 (1.61–2.21) | <0.001 | 1.16 (1.04–1.29) | 0.006 |
|  | Increase per 1-SD | 1.15 (1.13–1.18) | <0.001 | 1.28 (1.23–1.33) | <0.001 | 1.08 (1.04–1.11) | <0.001 |

**Notes:** Hazard ratios (HRs) and 95% confidence intervals (CIs) were estimated using competing risk models with all-cause mortality treated as a competing event. METS-VF and BRI were entered separately in Model 3 to avoid potential multicollinearity. Q1 was used as the reference group. Per 1-SD estimates represent the risk associated with each standard deviation increase in METS-VF or BRI.

**Abbreviations:** BRI, body roundness index; CI, confidence interval; eGFR, estimated glomerular filtration rate; HR, hazard ratio; METS-VF, metabolic score for visceral fat; Q1, quartile 1; SD, standard deviation; TDI, Townsend deprivation index; UA, uric acid.

**Table S7.** Sensitivity analysis of the associations between BRI quartiles and dementia risk after excluding incident dementia cases occurring within the first 5 years of follow-up

| **Variables** | **Overall** | **Quartiles of BRI** | | | |  |
| --- | --- | --- | --- | --- | --- | --- |
|  |  | **Q1 (≤3.18)** | **Q2 (3.18-4.07)** | **Q3 (4.07-5.12)** | **Q4 (>5.12)** | ***P for trend*** |
| **All-cause dementia** | | | | | | |
| Cases/total cases, n/N (%) | 8,085/326,685 (2.47) | 1,608/81,703 (1.97) | 1,826/81,680 (2.24) | 2,098/81,680 (2.57) | 2,553/81,622 (3.13) |  |
| per 1-SD  P-value | 1.16 (1.14, 1.19)  ＜0.001 | Ref (1.0) | 1.07 (1.00, 1.15)  0.058 | 1.19 (1.11, 1.27)  ＜0.001 | 1.44 (1.34, 1.54)  ＜0.001 | <0.001 |
| **Vascular dementia** | | | | | | |
| Cases/total cases, n/N (%) | 1,754/326,685 (0.54) | 260/81,703 (0.32) | 351/81,680 (0.43) | 451/81,680 (0.55) | 692/81,622 (0.85) |  |
| per 1-SD  P-value | 1.30 (1.24, 1.36)  ＜0.001 | Ref (1.0) | 1.17(1.00, 1.38)  0.056 | 1.38(1.18, 1.62)  ＜0.001 | 2.04 (1.74, 2.38)  ＜0.001 | <0.001 |
| **Alzheimer’s disease** | | | | | | |
| Cases/total cases, n/N (%) | 3,665/326,685 (1.12) | 812/81,703 (0.99) | 881/81,680 (1.08) | 947/81,680 (1.16) | 1025/81,622 (1.26) |  |
| per 1-SD  P-value | 1.09 (1.06, 1.13)  ＜0.001 | Ref (1.0) | 1.07 (0.97, 1.18)  0.202 | 1.13 (1.02, 1.25)  0.017 | 1.22 (1.10, 1.35)  ＜0.001 | ＜0.001 |

**Notes:** To mitigate potential reverse causation bias, participants who were diagnosed with incident dementia within the first 5 years of follow-up were excluded from this analysis. Hazard ratios (HRs) and 95% confidence intervals (CIs) were estimated using the fully adjusted Cox proportional hazards model (Model 3). Q1 was used as the reference group. The per 1-SD estimate represents the risk associated with each standard deviation increase in BRI.

**Abbreviations:** BRI, body roundness index; CI, confidence interval; eGFR, estimated glomerular filtration rate; HR, hazard ratio; Q1, quartile 1; Ref, reference; SD, standard deviation; TDI, Townsend deprivation index; UA, uric acid.

**Table S8. Sensitivity analysis of the associations between METS-VF quartiles and dementia risk after excluding incident dementia cases occurring within the first 5 years of follow-up**

| **Variables** | **Overall** | **Quartiles of** **METS-VF** | | | |  |
| --- | --- | --- | --- | --- | --- | --- |
|  |  | **Q1 (≤6.38)** | **Q2 (6.38-6.80)** | **Q3 (6.80-7.16)** | **Q4 (>7.16)** | ***P for trend*** |
| **All-cause dementia** | | | | | | |
| Cases/total cases, n/N (%) | 8,085/326,685 (2.47) | 1,537/81,710 (1.88) | 1,772/81,676 (2.17) | 2,125/81,664 (2.60) | 2,651/81,635 (3.25) |  |
| per 1-SD  P-value | 1.26 (1.22, 1.29)  ＜0.001 | Ref (1.0) | 1.09 (1.01, 1.17)  0.019 | 1.29 (1.21, 1.38)  ＜0.001 | 1.71 (1.59, 1.83)  ＜0.001 | <0.001 |
| **Vascular dementia** | | | | | | |
| Cases/total cases, n/N (%) | 1,754/326,685 (0.54) | 251/81,710 (0.31) | 338/81,676 (0.41) | 471/81,664 (0.58) | 694/81,635 (0.85) |  |
| per 1-SD  P-value | 1.51 (1.42, 1.60)  ＜0.001 | Ref (1.0) | 1.17(0.99, 1.38)  0.063 | 1.56 (1.33, 1.83)  ＜0.001 | 2.49 (2.13, 2.90)  ＜0.001 | <0.001 |
| **Alzheimer’s disease** | | | | | | |
| Cases/total cases, n/N (%) | 3,665/326,685 (1.12) | 756/81,710 (0.93) | 835/81,676 (1.02) | 966/81,664 (1.18) | 1108/81,635 (1.36) |  |
| per 1-SD  P-value | 1.18 (1.14, 1.23)  ＜0.001 | Ref (1.0) | 1.08 (0.98, 1.20)  0.115 | 1.25 (1.13, 1.38)  ＜0.001 | 1.46 (1.32, 1.61)  ＜0.001 | ＜0.001 |

**Notes:** To mitigate potential reverse causation bias, participants who were diagnosed with incident dementia within the first 5 years of follow-up were excluded from this analysis. Hazard ratios (HRs) and 95% confidence intervals (CIs) were estimated using the fully adjusted Cox proportional hazards model (Model 3). Q1 was used as the reference group. The per 1-SD estimate represents the risk associated with each standard deviation increase in METS-VF.

**Abbreviations:** BRI, body roundness index; CI, confidence interval; eGFR, estimated glomerular filtration rate; HR, hazard ratio; Q1, quartile 1; Ref, reference; SD, standard deviation; TDI, Townsend deprivation index; UA, uric acid.

**Table S9. Sensitivity analysis of the association between BRI and dementia risk after excluding participants with underweight at baseline (BMI < 18.5 kg/m²)**

| **Variables** | **Overall** | **Quartiles of BRI** | | | |  |
| --- | --- | --- | --- | --- | --- | --- |
|  |  | **Q1 (≤3.18)** | **Q2 (3.18-4.07)** | **Q3 (4.07-5.12)** | **Q4 (>5.12)** | ***P for trend*** |
| **All-cause dementia** | | | | | | |
| Cases/total cases, n/N (%) | 8,703/325,753 (2.67) | 1,686/80,246 (2.10) | 1,984/81,829 (2.42) | 2,260/81,836 (2.76) | 2,773/81,842 (3.39) |  |
| per 1-SD  P-value | 1.17 (1.15, 1.20)  ＜0.001 | Ref (1.0) | 1.09 (1.02, 1.16)  0.015 | 1.20 (1.12, 1.28)  ＜0.001 | 1.46 (1.36, 1.56)  ＜0.001 | <0.001 |
| **Vascular dementia** | | | | | | |
| Cases/total cases, n/N (%) | 1,946/325,753 (0.60) | 284/80,246 (0.35) | 399/81,829 (0.49) | 490/81,836 (0.60) | 773/81,842 (0.94) |  |
| per 1-SD  P-value | 1.29 (1.24, 1.35)  ＜0.001 | Ref (1.0) | 1.20 (1.02, 1.40)  0.024 | 1.35 (1.16, 1.57)  ＜0.001 | 2.05 (1.76, 2.37)  ＜0.001 | <0.001 |
| **Alzheimer’s disease** | | | | | | |
| Cases/total cases, n/N (%) | 3,927/325,753 (1.21) | 852/80,246 (1.06) | 950/81,829 (1.16) | 1,026/81,836 (1.25) | 1,099/81,842 (1.34) |  |
| per 1-SD  P-value | 1.09 (1.05, 1.13)  ＜0.001 | Ref (1.0) | 1.08 (0.98, 1.18)  0.132 | 1.15 (1.04, 1.26)  0.006 | 1.22 (1.11, 1.35)  ＜0.001 | ＜0.001 |

**Notes:** To reduce potential reverse causation bias related to preclinical disease-associated weight loss, participants with underweight at baseline (BMI < 18.5 kg/m²) were excluded from this analysis. Hazard ratios (HRs) and 95% confidence intervals (CIs) were estimated using the fully adjusted Cox proportional hazards model (Model 3). Q1 was used as the reference group. The per 1-SD estimate represents the risk associated with each standard deviation increase in BRI.

**Abbreviations:** BRI, body roundness index; BMI, body mass index; CI, confidence interval; eGFR, estimated glomerular filtration rate; HR, hazard ratio; Q1, quartile 1; Ref, reference; SD, standard deviation; TDI, Townsend deprivation index; UA, uric acid.

**Table S10. Sensitivity analysis of the association between METS-VF and dementia risk after excluding participants with underweight at baseline (BMI < 18.5 kg/m²)**

| **Variables** | **Overall** | **Quartiles of METS-VF** | | | |  |
| --- | --- | --- | --- | --- | --- | --- |
|  |  | **Q1 (≤3.18)** | **Q2 (3.18-4.07)** | **Q3 (4.07-5.12)** | **Q4 (>5.12)** | ***P for trend*** |
| **All-cause dementia** | | | | | | |
| Cases/total cases, n/N (%) | 8,703/325,753 (2.67) | 1,607/80,244 (2.00) | 1,935/81,830 (2.36) | 2,303/81,837 (2.81) | 2,858/81,842 (3.49) |  |
| per 1-SD  P-value | 1.28 (1.24, 1.31)  ＜0.001 | Ref (1.0) | 1.12 (1.04, 1.19)  0.001 | 1.32 (1.23, 1.41)  ＜0.001 | 1.73 (1.62, 1.85)  ＜0.001 | <0.001 |
| **Vascular dementia** | | | | | | |
| Cases/total cases, n/N (%) | 1,946/325,753 (0.60) | 273/80,244 (0.34) | 389/81,830 (0.48) | 522/81,837 (0.64) | 762/81,842 (0.93) |  |
| per 1-SD  P-value | 1.51 (1.43, 1.61)  ＜0.001 | Ref (1.0) | 1.22 (1.04, 1.43)  0.013 | 1.57 (1.35, 1.83)  ＜0.001 | 2.50 (2.15, 2.89)  ＜0.001 | <0.001 |
| **Alzheimer’s disease** | | | | | | |
| Cases/total cases, n/N (%) | 3,927/325,753 (1.21) | 787/80,244 (0.98) | 909/81,830 (1.11) | 1,043/81,837 (1.27) | 1,188/81,842 (1.45) |  |
| per 1-SD  P-value | 1.19 (1.15, 1.24)  ＜0.001 | Ref (1.0) | 1.11 (1.01, 1.23)  0.029 | 1.27 (1.16, 1.40)  ＜0.001 | 1.48 (1.34, 1.63)  ＜0.001 | ＜0.001 |

**Notes:** To reduce potential reverse causation bias related to preclinical disease-associated weight loss, participants with underweight at baseline (BMI < 18.5 kg/m²) were excluded from this analysis. Hazard ratios (HRs) and 95% confidence intervals (CIs) were estimated using the fully adjusted Cox proportional hazards model (Model 3). Q1 was used as the reference group. The per 1-SD estimate represents the risk associated with each standard deviation increase in METS-VF.

**Abbreviations:** BMI, body mass index; CI, confidence interval; eGFR, estimated glomerular filtration rate; HR, hazard ratio; METS-VF, metabolic score for visceral fat; Q1, quartile 1; Ref, reference; SD, standard deviation; TDI, Townsend deprivation index; UA, uric acid.

**Table S11. Associations of changes in METS-VF and BRI with risk of the study outcome across multivariable-adjusted models**

| **Indicator** | **Model** | **Changes** | **Cases/total, n (%)** | **HR (95% CI)** | **P value** |
| --- | --- | --- | --- | --- | --- |
| **METS-VF** | Model 1 | low–low | 78/5113 (1.53) | Ref |  |
|  |  | low–high | 21/1247 (1.68) | 1.11 (0.68, 1.79) | 0.683 |
|  |  | high–low | 13/649 (2.00) | 1.33 (0.74, 2.39) | 0.347 |
|  |  | high–high | 95/3940 (2.41) | 1.63 (1.21, 2.20) | 0.001 |
|  | Model 2 | low–low | 78/5113 (1.53) | Ref |  |
|  |  | low–high | 21/1247 (1.68) | 1.10 (0.68, 1.78) | 0.710 |
|  |  | high–low | 13/649 (2.00) | 1.31 (0.73, 2.36) | 0.366 |
|  |  | high–high | 95/3940 (2.41) | 1.55 (1.15, 2.11) | 0.004 |
|  | Model 3 | low–low | 78/5113 (1.53) | Ref |  |
|  |  | low–high | 21/1247 (1.68) | 0.94 (0.61, 1.46) | 0.795 |
|  |  | high–low | 13/649 (2.00) | 1.25 (0.57, 2.74) | 0.571 |
|  |  | high–high | 95/3940 (2.41) | 1.60 (1.16, 2.21) | 0.004 |
| **BRI** | Model 1 | low–low | 80/4792 (1.67) | Ref |  |
|  |  | low–high | 20/1437 (1.39) | 0.83 (0.51, 1.35) | 0.445 |
|  |  | high–low | 8/640 (1.25) | 0.75 (0.37, 1.56) | 0.447 |
|  |  | high–high | 99/4080 (2.43) | 1.50 (1.12, 2.02) | 0.007 |
|  | Model 2 | low–low | 80/4792 (1.67) | Ref |  |
|  |  | low–high | 20/1437 (1.39) | 0.77 (0.47, 1.27) | 0.306 |
|  |  | high–low | 8/640 (1.25) | 0.71 (0.34, 1.48) | 0.363 |
|  |  | high–high | 99/4080 (2.43) | 1.32 (0.97, 1.79) | 0.075 |
|  | Model 3 | low–low | 80/4792 (1.67) | Ref |  |
|  |  | low–high | 20/1437 (1.39) | 0.81 (0.52, 1.26) | 0.347 |
|  |  | high–low | 8/640 (1.25) | 0.43 (0.14, 1.38) | 0.158 |
|  |  | high–high | 99/4080 (2.43) | 1.39 (1.00, 1.94) | 0.048 |

**Notes:** HRs and 95% CIs were estimated using Cox proportional hazards models for the associations of changes in METS-VF and BRI with the risk of the study outcome. Change patterns were classified as low-low, low-high, high-low, and high-high, with low-low as the reference. Model 1 was unadjusted; Model 2 was adjusted for age, sex, ethnicity, TDI, educational level, smoking status, and alcohol consumption; and Model 3 was further adjusted for dietary pattern, physical activity level, sleep duration, UA, eGFR, insulin use, aspirin use, antihypertensive drug use, and cholesterol-lowering medications. METS-VF and BRI were analyzed separately.

**Abbreviations:** BRI, body roundness index; CI, confidence interval; eGFR, estimated glomerular filtration rate; HR, hazard ratio; METS-VF, metabolic score for visceral fat; Ref, reference; TDI, Townsend deprivation index; UA, uric acid.

**Table S12. Sensitivity analysis of METS-VF and BRI change trajectories after excluding incident dementia cases within 5 years after the second follow-up**

| **Indicator** | **Model** | **Changes** | **Cases/total, n (%)** | **HR (95% CI)** | **P value** |
| --- | --- | --- | --- | --- | --- |
| **METS-VF** | Model 1 | low–low | 59/4405 (1.34) | Ref |  |
|  |  | low–high | 24/1932 (1.24) | 0.93 (0.58, 1.49) | 0.747 |
|  |  | high–low | 7/378 (1.85) | 1.40 (0.64, 3.07) | 0.400 |
|  |  | high–high | 80/4183 (1.91) | 1.47 (1.05, 2.06) | 0.024 |
|  | Model 2 | low–low | 59/4405 (1.34) | Ref |  |
|  |  | low–high | 24/1932 (1.24) | 0.91 (0.56, 1.46) | 0.690 |
|  |  | high–low | 7/378 (1.85) | 1.39 (0.64, 3.05) | 0.408 |
|  |  | high–high | 80/4183 (1.91) | 1.42 (1.01, 1.99) | 0.046 |
|  | Model 3 | low–low | 59/4405 (1.34) | Ref |  |
|  |  | low–high | 24/1932 (1.24) | 0.95 (0.59, 1.52) | 0.815 |
|  |  | high–low | 7/378 (1.85) | 1.49 (0.68, 3.28) | 0.324 |
|  |  | high–high | 80/4183 (1.91) | 1.55 (1.08, 2.21) | 0.017 |
| **BRI** | Model 1 | low–low | 63/4248 (1.48) | Ref |  |
|  |  | low–high | 22/1959 (1.12) | 0.75 (0.46, 1.22) | 0.253 |
|  |  | high–low | 3/408 (0.74) | 0.50 (0.16, 1.58) | 0.235 |
|  |  | high–high | 82/4283 (1.91) | 1.34 (0.96, 1.86) | 0.081 |
|  | Model 2 | low–low | 63/4248 (1.48) | Ref |  |
|  |  | low–high | 22/1959 (1.12) | 0.72 (0.44, 1.17) | 0.184 |
|  |  | high–low | 3/408 (0.74) | 0.48 (0.15, 1.52) | 0.209 |
|  |  | high–high | 82/4283 (1.91) | 1.21 (0.86, 1.70) | 0.279 |
|  | Model 3 | low–low | 63/4248 (1.48) | Ref |  |
|  |  | low–high | 22/1959 (1.12) | 0.74 (0.45, 1.20) | 0.221 |
|  |  | high–low | 3/408 (0.74) | 0.49 (0.15, 1.55) | 0.224 |
|  |  | high–high | 82/4283 (1.91) | 1.30 (0.91, 1.87) | 0.149 |

**Notes:** HRs and 95% CIs were estimated using Cox proportional hazards models. To reduce potential prodromal reverse causation, participants who developed incident dementia within 5 years after the second follow-up were excluded. Change trajectories were classified as low-low, low-high, high-low, and high-high according to baseline and follow-up levels, with the low-low group as the reference. Model 1 was unadjusted; Model 2 was adjusted for age, sex, ethnicity, Townsend deprivation index, educational level, smoking status, and alcohol consumption; and Model 3 was further adjusted for dietary pattern, physical activity level, sleep duration, uric acid, estimated glomerular filtration rate, insulin use, aspirin use, antihypertensive drug use, and cholesterol-lowering medications. METS-VF and BRI were analyzed separately.

**Abbreviations:** BRI, body roundness index; CI, confidence interval; HR, hazard ratio; METS-VF, metabolic score for visceral fat.

**Table S13. Incremental explanatory contribution of METS-VF and BRI beyond BMI- or WC-based models for dementia risk**

| **Variable** | **Model** | **Indicator** | **Nagelkerke *R*^2^** | **ΔR²** | **HR（95% CI）** | **P value** |
| --- | --- | --- | --- | --- | --- | --- |
| BMI | Model 1 | BMI residual | 0.2913 | Ref | - | - |
|  | Model 2 | BMI residual + METS-VF | 0.2947 | 0.0034 | 1.62 (1.58, 1.66） | <0.001 |
|  | Model 3 | BMI residual + BRI | 0.2949 | 0.0036 | 1.57 (1.54, 1.60） | <0.001 |
| WC | Model 1 | WC residual | 0.2565 | Ref | - | - |
|  | Model 2 | WC residual + METS-VF | 0.2698 | 0.0133 | 2.87 (2.79, 2.96） | <0.001 |
|  | Model 3 | WC residual+ BRI | 0.2726 | 0.0161 | 2.74 (2.69, 2.80） | <0.001 |

**Notes:** To quantify the explanatory contribution of METS-VF and BRI beyond traditional obesity indices, two-step analyses were performed. First, Cox proportional hazards models including BMI alone or WC alone were fitted, and Martingale residuals were extracted to represent dementia risk variation not explained by BMI or WC. Second, these residuals were jointly entered with METS-VF or BRI into secondary Cox models. The incremental explanatory contribution of METS-VF or BRI was quantified by the change in Nagelkerke pseudo-R² (ΔR²). Hazard ratios (HRs) and 95% confidence intervals (CIs) were estimated to assess the independent associations of METS-VF and BRI with dementia risk.

**Abbreviations:** BRI, body roundness index; CI, confidence interval; HR, hazard ratio; METS-VF, metabolic score for visceral fat; WC, waist circumference.

**Table S14. Shapiro-Wilk normality test of anthropometric and metabolic markers among participants who subsequently developed dementia**

| Variable | Shapiro–Wilk W | P value |
| --- | --- | --- |
| BMI | 0.95 | <0.001 |
| WC | 0.99 | 0.004 |
| FBG | 0.68 | <0.001 |
| TG | 0.83 | <0.001 |
| HDL-C | 0.97 | <0.001 |

**Notes:** Shapiro-Wilk tests were performed to assess the normality of anthropometric and metabolic markers measured at baseline and follow-up among participants who subsequently developed dementia. Because all variables showed significant deviation from normality (*P* < 0.05), nonparametric paired comparisons were used in subsequent analyses.

**Abbreviations:** BMI, body mass index; FBG, fasting blood glucose; HDL-C, high-density lipoprotein cholesterol; TG, triglycerides; WC, waist circumference.

**Table S15. Changes in anthropometric and metabolic markers from baseline to first follow-up among participants who subsequently developed dementia**

| **Variable** | **Baseline** | **Follow-up** | **Statistic value** | **Z value** | **P value** |
| --- | --- | --- | --- | --- | --- |
| n | 207 | 207 |  |  |  |
| WC | 92 (80, 100) | 92 (84, 102) | 6003.5 | 5.52 | <0.001 |
| BMI | 26.42 (24.00, 29.58) | 26.42 (23.63, 29.69) | 11478.5 | 0.83 | 0.408 |
| FBG | 4.91 (4.56, 5.38) | 5.07 (4.76, 5.47) | 7775.0 | 3.46 | 0.001 |
| TG | 1.44 (1.04, 2.05) | 1.39 (0.97, 1.94) | 12519.5 | 2.03 | 0.042 |
| HDL-C | 1.38 (1.12, 1.72) | 1.48 (1.17, 1.79) | 7167.0 | 4.17 | <0.001 |

**Notes:** Baseline and first follow-up anthropometric and metabolic markers were compared among participants who subsequently developed dementia. Because all variables were non-normally distributed according to the Shapiro-Wilk test, data are presented as median (interquartile range [IQR]) and were compared using the Wilcoxon signed-rank test. The statistic value refers to the Wilcoxon signed-rank test statistic, and the corresponding Z and *P* values are reported.

**Abbreviations:** BMI, body mass index; FBG, fasting blood glucose; HDL-C, high-density lipoprotein cholesterol; IQR, interquartile range; TG, triglycerides; WC, waist circumference.

**Figure S1.** LASSO-Cox regression analyses for metabolite selection in all-cause dementia, vascular dementia, and Alzheimer’s disease


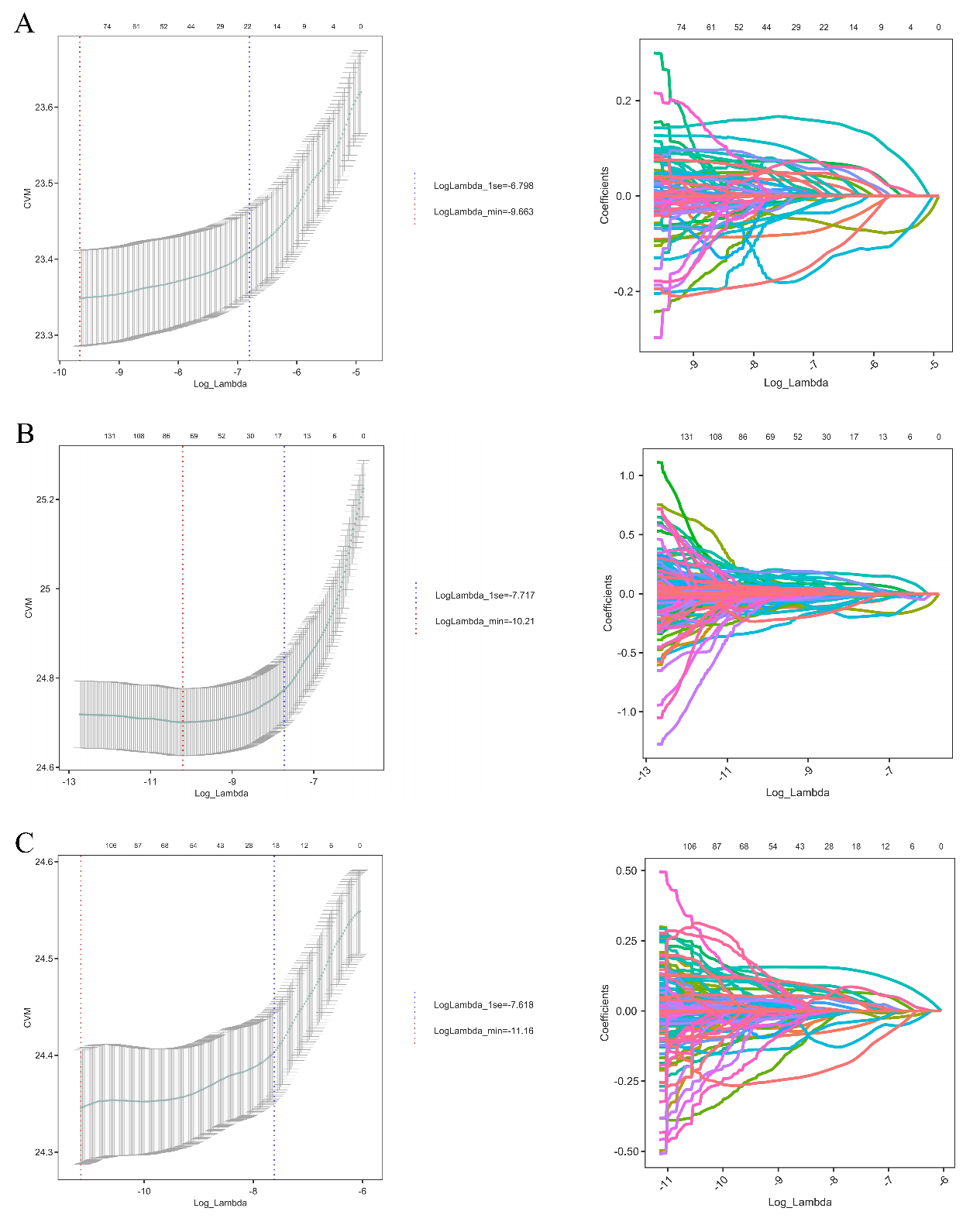


(A–C) show the LASSO-Cox analyses for all-cause dementia, vascular dementia, and Alzheimer’s disease, respectively. In each panel, the left subplot shows the relationship between the cross-validated mean error (CVM) and log(lambda), with the gray band indicating the standard error and the dashed lines indicating lambda.min and lambda.1se. The right subplot shows the coefficient trajectories of metabolites across log(lambda), illustrating the shrinkage process used for metabolite selection.

**Figure S2.** Proposed pathway linking visceral obesity, metabolic dysfunction, and dementia risk.


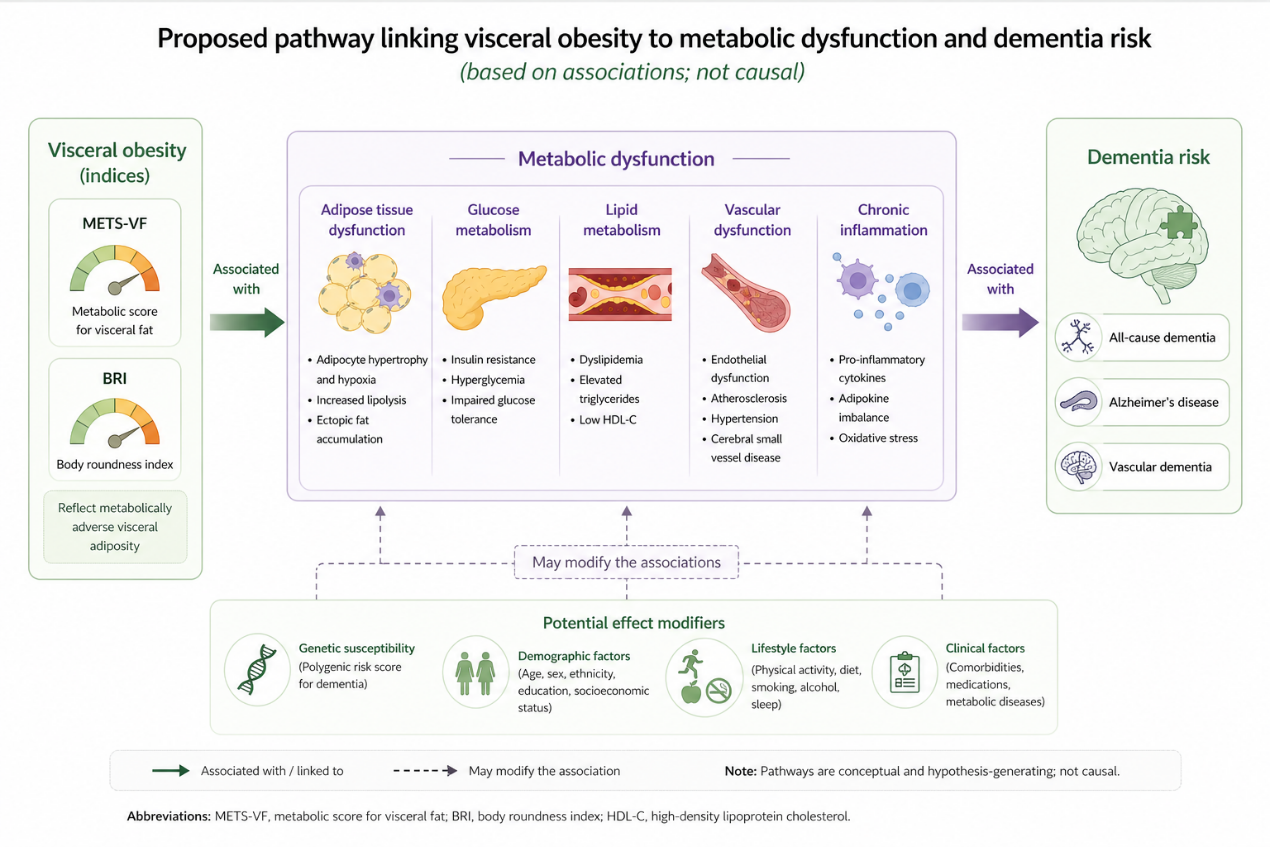


This schematic illustrates the potential associations between visceral obesity indices (METS-VF and BRI), metabolic dysregulation, and dementia risk. Visceral adiposity is linked to adverse metabolic outcomes, including insulin resistance, dyslipidemia, and inflammation, which may increase the risk of vascular and neurodegenerative injuries. The figure highlights the role of genetic susceptibility and other modifying factors in these associations. The pathways depicted are conceptual and hypothesis-generating, based on associations observed in this study.
